# The Efficacy and Safety of Bedaquiline in the Treatment of Pulmonary Tuberculosis Patients: A Systematic Review and Meta-Analysis

**DOI:** 10.3390/antibiotics12091389

**Published:** 2023-08-31

**Authors:** Enyu Tong, Qian Wu, Yiming Chen, Zhengwei Liu, Mingwu Zhang, Yelei Zhu, Kunyang Wu, Junhang Pan, Jianmin Jiang

**Affiliations:** 1 School of Public Health, Hangzhou Normal University, Hangzhou 311100, China; 2 Zhejiang Provincial Center for Disease Control and Prevention, Hangzhou 310051, China; 3 Key Lab of Vaccine, Prevention and Control of Infectious Disease of Zhejiang Province, Hangzhou 310051, China

**Keywords:** efficacy, safety, bedaquiline, pulmonary tuberculosis, systematic review, meta-analysis

## Abstract

Background: Bedaquiline (BDQ) has been designated as a Group A drug by the World Health Organization (WHO) for the management of multi-drug-resistant tuberculosis (MDR-TB) and extensively drug-resistant tuberculosis (XDR-TB). This systematic review and meta-analysis aim to evaluate the efficacy and safety of BDQ-containing regimens for the treatment of patients with pulmonary TB. Methods: PubMed (MEDLINE), Elton B. Stephens Company (EBSCO) database, the Cochrane Register of Controlled Trials, and the China National Knowledge Infrastructure (CNKI) database were initially searched on 15 June 2022 and again on 20 March 2023. We included randomized controlled trials (RCTs) and non-randomized studies (NRSs) that administered BDQ to TB patients. The outcomes of interest were as follows: (1) efficacy, including the rate of sputum culture conversion at 8 weeks, 24 weeks, and during follow-up, as well as the rates of completion cure, death, treatment failure, and loss at follow-up and at the end of the treatment; and (2) safety, which encompassed the incidences of cardiotoxicity, hepatotoxicity, and grade 3–5 adverse events during the treatment period. Results: A total of 29 articles were included in this meta-analysis, representing 23,358 individuals. Patients who were treated with BDQ were compared with patients who were not exposed to BDQ. The use of BDQ-containing regimens demonstrated improved rates of sputum conversion in RCTs at 24 weeks (RR = 1.27, 95% CI: 1.10 to 1.46) and during follow-up (RR = 1.33, 95% CI: 1.06 to 1.66). Additionally, BDQ-containing regimens showed increased cure rates (RR = 1.60, 95% CI: 1.13 to 2.26) and decreased failure rates (RR = 0.56, 95% CI: 0.56 to 0.88). In NRSs, BDQ-containing regimens improved the sputum culture conversion rate during follow-up (RR = 1.53, 95% CI: 1.07 to 2.20), increased the rate of cure (RR = 1.86, 95% CI: 1.23 to 2.83), reduced deaths from all causes (RR = 0.68, 95% CI: 0.48 to 0.97), and reduced failure rates (RR = 0.57, 95% CI: 0.46 to 0.71). However, the use of BDQ-containing regimens was associated with increased incidences of cardiotoxicity (RR = 4.54, 95% CI: 1.74 to 11.87) and grade 3–5 adverse events (RR = 1.42, 95% CI: 1.17 to 1.73) in RCTs. NRSs also showed an association between BDQ-containing regimens and cardiotoxicity (RR = 6.00, 95% CI: 1.32 to 27.19). No significant differences were observed between intervention groups and control groups with respect to other outcomes. Conclusions: Data from both RCTs and NRSs support the efficacy of BDQ for the treatment of pulmonary tuberculosis. However, the use of BDQ is associated with a higher incidence of cardiotoxicity and serious adverse events. Comparative data on efficacy and safety are limited, and further confirmation is required, due to potential bias and discrepancies in the available studies.

## 1. Introduction

Tuberculosis (TB) is a chronic infectious disease caused by *Mycobacterium tuberculosis* (MTB). The World Health Organization (WHO) has estimated that a quarter of the world’s population is infected with MTB [1]. In 2020, approximately 7.1 million new TB cases were diagnosed and reported, resulting in approximately 1.52 million deaths. Among these deaths, an estimated 1.3 million occurred in people without human immunodeficiency virus (HIV) infection, while an additional 214,000 deaths were reported in HIV-positive individuals [1].

Rifampicin-resistant TB (RR-TB) refers to MTB strains that are resistant to rifampicin, while multi-drug-resistant tuberculosis (MDR-TB) is caused by bacteria that are resistant, at least, to isoniazid and rifampicin. The Global TB Report 2021 estimated that 132,222 cases of MDR-TB and RR-TB were detected in 2020. The emergence of MDR-TB and RR-TB poses a significant threat to TB control and has become an increasing concern for global public health [1]. As a result, the management of TB infection is a crucial priority under the global “End TB” strategy [1].

Bedaquiline (BDQ), previously known as TMC207 or R207910, is a novel anti-TB drug that belongs to the class of compounds called diarylquinolines [2,3]. This drug exhibits a potent inhibitory effect on several species of mycobacteria by targeting adenosine triphosphate (ATP) synthase, a crucial enzyme that is responsible for energy production in TB, especially MTB [3]. Its mechanism involves obstructing the ion-binding sites of mycobacterial ATP synthase, which is responsible for converting ADP to ATP using a transmembrane electrochemical ion (H^+^ or Na^+^) gradient to generate energy. Specifically, BDQ selectively targets the ion-binding sites found in the c-subunit of ATP synthase, impeding the proton pump and leading to a reduction in intracellular ATP levels, which are essential for energy production in tuberculosis [3,4]. 

Following promising results from Phase II and Phase III trials, BDQ received approval from the US Food and Drug Administration (FDA) in 2012 as a Group A drug for the treatment of MDR-TB [5,6], and by the end of 2019, 109 countries had reported importing or initiating the use of BDQ as a first-line drug for MDR-TB patients [6]. In the same year, four nations—India, South Africa, the Russian Federation, and Ukraine—accounted for 68% of the globally treated patients receiving BDQ [7].

In December 2022, WHO issued the latest treatment guidelines for MDR-TB and RR-TB, recommending a 6-month treatment regime that includes BDQ, pretomanid, linezolid (LZD), and moxifloxacin (BPaLM). The suggested dosage for BDQ is 400 mg daily for the first two weeks, followed by 200 mg three times a week for the next 22 weeks. To enhance efficiency and absorption, WHO advises taking the medication with food. This new treatment regimen is designed to replace the previously used 9-month or 18-month regimens [8]. Despite BDQ’s potential as a very promising anti-TB drug, there are still concerns regarding its efficacy and safety [1].

Therefore, we conducted a systematic review and meta-analysis to pool and analyse the efficacy and safety-related outcomes of BDQ-containing treatment regimens in pulmonary TB (PTB) patients.

## 2. Results

### 2.1. Study Selection and Characteristics

**Figure 1:**
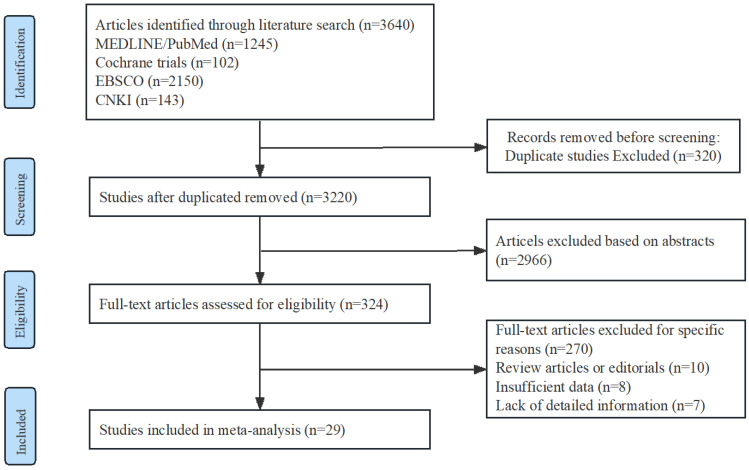
Flow diagram of study identification, screening, eligibility assessment, and inclusion.

A Preferred Reporting Items for Systematic Reviews and Meta-analyses flow chat is shown in Figure 1. Among the included studies, there were 12 randomized controlled trials (RCTs) with a total of 1079 participants, and 17 non-randomized studies (NRSs) with a total of 22,279 participants. Among the NRSs, 5 were prospective and 12 were retrospective in design. Detailed information regarding the trials and characteristics of the patients can be found in Table 1.

The RCTs were mainly conducted in South Africa and China; the two remaining studies were multicentre. According to the definitions published by the WHO [9], these countries have high TB, TB/HIV, and MDR-TB burdens. The NRSs were conducted in various locations. Six studies were based in South Africa, six in China, and one in Papua New Guinea. These countries also experience a high burden of TB, TB/HIV, and MDR-TB. Two studies were conducted in the United States and South Korea, which have lower TB burden and MDR-TB burden. Two studies were conducted in Europe, and they were included on the list of priority attention for TB at the regional level [9].

**Table 1:** Characteristics of included studies.

First Author and Study Year	Study Design	Recruitment Date	Setting	Population	Sample Size	Regimens and Arms Used	Duration of Treatment
Diacon et al., 2009 [10]	RCT	June 2007–January 2008	A hospital, South Africa	Adults, MDR-TB, treatment-naive, pulmonary sputum smear-positive; Excl. HIV-positive on ART or CD4 < 300 cells/μL.	44	Arm 1: BR + Bdq Arm 2: BR + placebo	8 weeks
Diacon et al., 2012 [11]	RCT	N. S	A hospital, South Africa	Adults, MDR-TB; pulmonary sputum smear-positive; Excl. HIV-positive on ART or CD4 < 300 cells/μL.	44	Arm 1: BR + Bdq Arm 2: BR + placebo	104 weeks
Diacon et al., 2012 [12]	RCT	October 2010–August 2011	A hospital, South Africa	Adults, treatment-naive, pulmonary Rs-TB; Excl. HIV-positive on ART or CD4 ≤ 300 cells/μL.	85	Arm 1: Pa + Bdq Arm 2: Bdq Arm 3: Bdq + Z Arm 4: Pa + Z Arm 5: Pa + Mfx + Z Arm 6: HRZE	2 weeks
Diacon et al., 2014 [13]	RCT	June 2007–March 2010	Brazil, India, Latvia, Peru, the Philippines, Russia, South Africa, Thailand	Adults, MDR-TB, treatment-naive, pulmonary sputum smear-positive; Excl. HIV-positive on CD4 < 300 cells/μL.	160	Arm 1: BR + Bdq Arm 2: BR + placebo	120 weeks
Diacon et al., 2015 [14]	RCT	October 2012–April 2013	Outpatient clinics in Cape Town, South Africa	Adults, treatment-naive, pulmonary sputum smear-positive Rs and Hs-TB; Excl. HIV-positive with CD4 ≤ 300 cells/μL.	105	Arm 1: Bdq + Pa + Z + C Arm 2: Bdq + Pa + Z Arm 3: Bdq + Pa + C Arm 4: Bdq + Z + C Arm 5: Z Arm 6:C Arm 7: HRZE	2 weeks
Esmail et al., 2022 [15]	RCT	November 2015–December 2020	Five centres, South Africa	Adults, treatment-naive, MDR/RR-TB, pulmonary sputum smear-positive; Excl. XDR-TB, pre-XDR-TB.	111	Arm 1: BR + Bdq Arm 2: BR	96 weeks
Ling et al., 2021 [16]	RCT	September 2018–January 2020	A hospital, China	Adults, MDR-TB; Excl. HIV-positive.	64	Arm 1: BR + Bdq Arm 2: BR	24 weeks
Mou. 2021 [17]	RCT	February 2019–May 2020	A hospital, China	Adults, MDR-TB.	63	Arm 1: BR + Bdq Arm 2: BR + placebo	72 weeks
Ren et al., 2021 [18]	RCT	December 2017–June 2019	A hospital, China	Adults, MDR-TB; Excl. HIV-positive, HBV-positive.	60	Arm 1: BR + Bdq Arm 2: BR	24 weeks
Tweed et al., 2019 [19]	RCT with single Rr-TB arm	October 2014–February 2016	South Africa, Tanzania, Uganda	Adults, RR-TB; treatment-naive pulmonary, sputum smear-positive Rs-TB; Excl. HIV-positive with CD4 < 200 cells/mL.	240	Arm 1: Bdq + Pa + Z Arm 2: Bdq 200 + Pa + Z Control: HRZE	8 weeks
Wang 2019 [20]	RCT	March 2019–August 2020	A hospital, China	Adults, MDR-TB.	69	Arm 1: BR + Bdq Arm 2: BR	26 weeks
Wu 2020 [21]	RCT	January 2018–July 2018	A hospital, China	Adults, MDR-TB; sputum smear-positive.	34	Arm 1: BR + Bdq Arm 2: BR	24 weeks
Fu et al., 2021 [22]	PCS	April 2019–August 2020	A hospital, China	DR/MDR-TB Excl. HIV-positive, AIDS.	103	Arm 1: BR + Bdq Arm 2: BR	16 weeks
Chang et al., 2021 [23]	PCS	January 2018–June 2019	A hospital, China	Adults, MDR-TB.	90	Arm 1: BR + Bdq Arm 2: BR	24 weeks
Kempker et al., 2020 [24]	PCS	December 2015–May 2017	A centre, Georgia, USA	Adults, Smear positive MDR-TB.	95	Arm 1: BR + Bdq Arm 2: BR + DLM	24 weeks
Kim et al., 2018 [25]	PCS	January 2015–October 2017	Hospitals, South Korea	Adults, MDR-TB.	50	Arm 1: BR + Bdq Arm 2: BR + DLM	24 weeks
Olayanju et al., 2018 [26]	PCS	January 2008–June 2017	A hospital, South Africa	Adults, XDR-TB.	272	Arm 1: BR + Bdq Arm 2: BR	96 weeks
Chen et al., 2021 [27]	RCS	June 2016–August 2019	A centre, China	Adults, RR/MDR-TB.	112	Arm 1: BR + Bdq Arm 2: BR	24 weeks
Chesov et al., 2021 [28]	RCS	2016–2018	the Republic of Moldova	Adults, sputum smear-positive MDR-TB.	228	Arm 1: BR + Bdq Arm 2: BR	24 weeks
Bastard et al., 2018 [29]	RCS	September 2005-April 2015	Armenia	Adults, MDR-TB	140	Arm 1: BR + Bdq Arm 2: BR	24 weeks
Kang et al., 2020 [30]	RCS	September 2016–February 2018;	A centre, South Korea	MDR-TB	215	Arm 1: BR + Bdq Arm 2: BR + DLM	d48 weeks
Liu et al., 2021 [31]	RCS	January 2017–January 2018	A hospital, China	Adults, RR/MDR-TB	174	Arm 1:BR + Bdq Arm 2:BR	96 weeks
Padayatchi et al., 2020 [32]	RCS	January 2014–November 2015	A hospital, South Africa	Adults, DR-TB	256	Arm 1: BR + Bdq Arm2: BR	24 weeks
Ren et al., 2021 [33]	RCS	June 2018–January 2020	A hospital, China	Adults, MDR-TB	60	Arm 1: BR + Bdq Arm 2: BR	24 weeks
Schnippel et al., 2018 [34]	RCS	July 2014–March 2016;	EDRweb, South Africa	Adults, DR-TB	19,617	Arm 1: BR + Bdq Arm 2: BR + SLID	72 weeks
Taune et al., 2019 [35]	RCS	June 2015–December 2017	Papua New Guinea	Adults, RR/MDR-TB	277	Arm 1: BR + Bdq Arm 2: BR	24 weeks
Zhang et al., 2022 [36]	RCS	November 2018–December 2020	A centre, China	Adults, RR/MDR-TB	127	Arm 1: BR + Bdq Arm 2: BR	24 weeks
Zhao et al., 2019 [37]	RCS	October 2014–October 2016	EDRweb, South Africa	Adults, MDR-TB Excl. pre-XDR-TB, XDR-TB	330	Arm 1: BR + Bdq Arm 2: BR	48 weeks
Hwang et al., 2021 [38]	RCS	September 2016–February 2018	A hospital, South Africa	Adults, MDR-TB	260	Arm 1: BR + Bdq Arm 2: BR + DLM	24 weeks

ART, anti-retroviral treatment; CD4, CD4 T-lymphocytes count; TB, tuberculosis; Hs-TB, isoniazid-susceptible tuberculosis; Rs, rifampicin-susceptible; Rr-TB, rifampicin-resistant tuberculosis; DR-TB, drug-resistant tuberculosis; MDR-TB, multidrug-resistant tuberculosis; pre-XDR-TB, pre-extensively resistant tuberculosis; BR, Background regimen; Bdq, bedaquiline; Mfx, moxifloxacin; Pa, pretomanid; SLID, second-line injectable drug; DLM, delamanid; HRZE, Isoniazid, Rifampicin, Pyrazinamide, Ethambutol; Z, pyrazinamide; RCT, randomized controlled trial; RCS, retrospective cohort study; PCS, prospective cohort study; Z, pyrazinamide; EDRweb, web-based electronic drug-resistant tuberculosis register.

### 2.2. Risk of Bias

**Figure 2:**
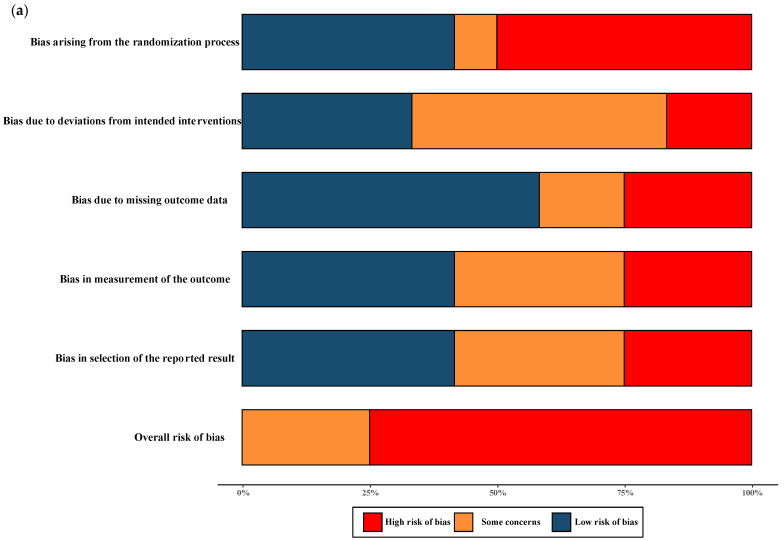

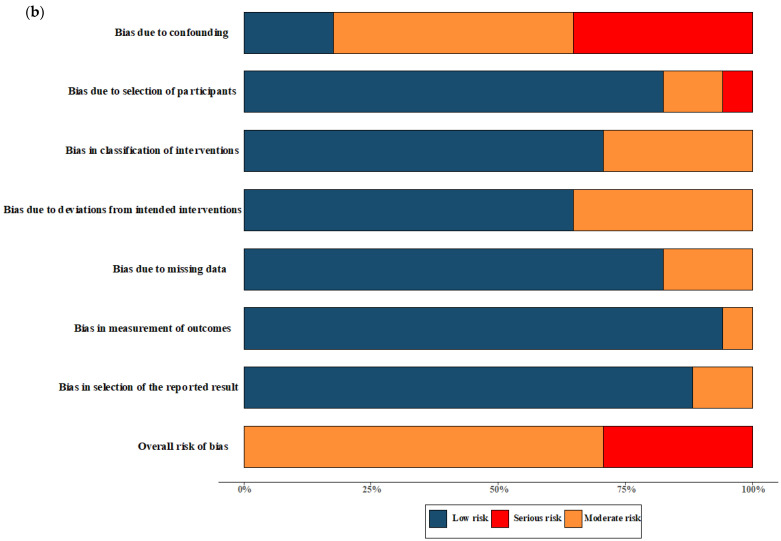
Risk of bias assessment. (**a**) Risk of bias in included randomised controlled trials. The colours in the bar next to each row/criteria represent the percentage of total studies falling within the high risk of bias/some concerns/low risk of bias. (**b**) Risk of bias in included non-randomised studies. The colours in the bar next to each row/criteria represent the percentage of total studies falling within the serious risk/moderate risk/low risk.

The overall quality of RCTs included in the meta-analysis were classified as having moderate-to-high risk of bias. All NRSs reporting primary and secondary outcomes were considered to be at moderate and severe risk of bias. The results of the “Risk of bias assessment” of the included RCTs and NRSs are summarized in Figure 2 (details of ROB 2.0 assessment and ROBINS-I assessment in each trial are shown in Figure S1).

The quality assessment of the RCTs included in the meta-analysis revealed a classification of moderate-to-high risk of bias. For the NRSs reporting both primary and secondary outcomes, a moderate-to-severe risk of bias was observed, and no critical risk was observed. The comprehensive results of the risk of bias assessment for both RCTs and NRSs can be found in Figure 2. Further details regarding the assessment using the ROB 2.0 tool for RCTs and the ROBINS-I tool for NRSs in each trial are presented in Figure S1.

### 2.3. Efficacy of Interventions

#### 2.3.1. The Rate of Sputum Culture Conversion

##### The Rate of Sputum Culture Conversion at 8 Weeks

Seven studies with 575 participants were included [10,11,15,22,24,27,30] and compared the rate of culture conversion in participants taking different regimens at 8 weeks, three of which were RCTs (Figure S2). The RCTs showed no significant difference in culture conversion rates between those who initiated the BDQ-containing regimen (RR = 1.48, 95% CI: 0.79 to 2.77). Four NRSs also revealed no significant difference in any of the treatments (RR = 1.07, 95% CI: 0.87 to 1.32).

##### The Rate of Sputum Culture Conversion at 24 Weeks

In 2013, Centers for Disease Control and Prevention (CDC) issued interim recommendations for the use of BDQ [39]. The CDC recommended that treatment over 24 weeks should be considered on a case-by-case basis, unless another effective treatment option is available [40]. As suggested by the WHO protocol, BDQ should not be prescribed for over 24 weeks [41]. Therefore, we estimated the effect of sputum culture conversion rate when BDQ was added to the background regimen at 24 weeks.

**Figure 3:**
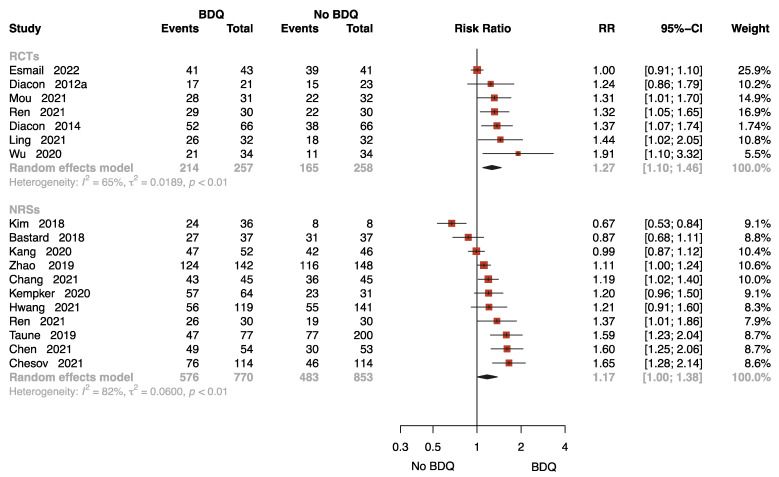
Forest plot of the rate of sputum culture conversion at 24 weeks. BDQ: bedaquiline; RCTs: randomized controlled trials; NRSs: non-randomized studies; RR: relative risks; CI: confidence interval. If heterogeneity *I*^2^ < 50% and *p*-value > 0.01, we used a fixed effects model; if heterogeneity *I*^2^ > 50% or *p*-value < 0.01, we used a random effects model [11,13,15,16,17,18,21,23,24,25,27,28,29,30,33,35,37,38].

Eighteen studies with 2138 patients provided data on sputum culture conversion rate at 24 weeks [11,13,15,16,17,18,21,23,24,25,27,28,29,30,33,35,37,38], seven of which were RCTs. The results of the RCTs showed that the BDQ-containing regimen reduced the rate of sputum-culture-positive compared to the no-BDQ-containing regimens (RR = 1.27, 95% CI: 1.10 to 1.46). The results found no significant difference between the intervention and comparator in NRSs (RR = 1.17, 95% CI: 1.00 to 1.38) (Figure 3).

The funnel plot for estimating publication bias for NRSs was roughly symmetrical (Figure S3). No publication bias was detected with Egger’s test for the NRSs (*p* = 0.360). No funnel plot of RCTs has been included as there were fewer than 10 studies.

##### The Rate of Sputum Culture Conversion with Follow-Up

**Figure 4:**
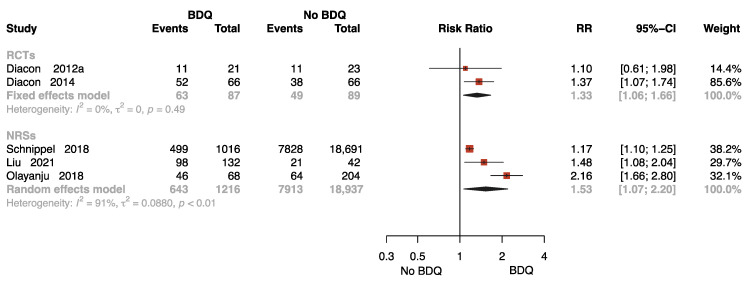
Forest plot of the rate of sputum culture conversion with follow up. BDQ: bedaquiline; RCTs: randomized controlled trials; NRSs: non-randomized studies; RR: relative risks; CI: confidence interval. If heterogeneity *I*^2^ < 50% and *p*-value > 0.01, we used a fixed effects model; if heterogeneity *I*^2^ > 50% or *p*-value < 0.01, we used a random effects model [11,13,26,31,34].

Five studies with 20,329 participants reported results on sputum culture conversion rates with follow-up [11,13,26,31,34], two of which were RCTs. One study was followed up for 72 weeks [34], two studies for 96 weeks [26,31], and two studies for 104 [11] and 120 weeks [13], respectively. As presented in Figure 4, the pooled data show that BDQ-containing regimen administration resulted in a significant increase in culture conversion rate compared to no-BDQ regimens both in RCTs (RR = 1.33, 95% CI: 1.06 to 1.66) and NRSs (RR = 1.53, 95% CI: 1.07 to 2.20).

#### 2.3.2. The Other Treatment Outcomes

##### The Rate of Complete at End of the Treatment

This was reported by fourteen studies with 21,515 participants [11,13,15,18,21,23,24,26,27,29,30,32,34,38], five of which were RCTs. The meta-analysis found no significant difference between the intervention groups and control groups (Figure S4).

##### The Rate of Cure at End of the Treatment

**Figure 5:**
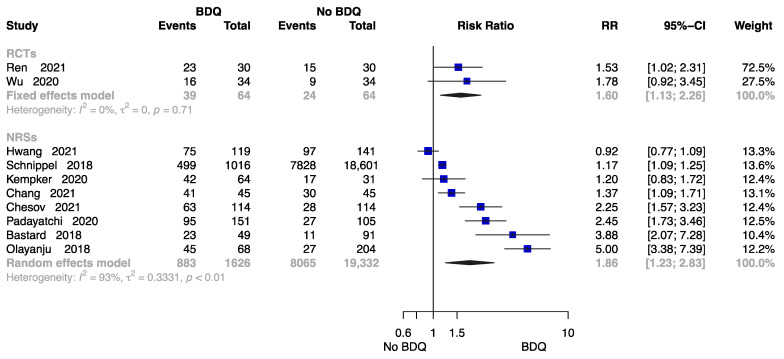
Forest plot of the rate of cure at end of treatment. BDQ: bedaquiline; RCTs: randomized controlled trials; NRSs: non-randomized studies; RR: relative risks; CI: confidence interval. If heterogeneity *I*^2^ < 50% and *p*-value > 0.01, we used a fixed effects model; if heterogeneity *I*^2^ > 50% or *p*-value < 0.01, we used a random effects model [18,21,23,24,26,28,29,32,34,38].

Figure 5 summarizes the findings from 10 trials with 21,086 patients [18,21,23,24,26,28,29,32,34,38], two of which were RCTs. Compared to those undergoing a no-BDQ-containing regimen, those who received a BDQ-containing regimen had a higher rate of treatment cure (RCTs: RR = 1.60, 95% CI 1.13 to 2.26; NRSs: RR = 1.86, 95% CI 1.23 to 2.83). The results are shown in Figure 5.

##### The Rate of All-Cause Death at End of the Treatment

**Figure 6:**
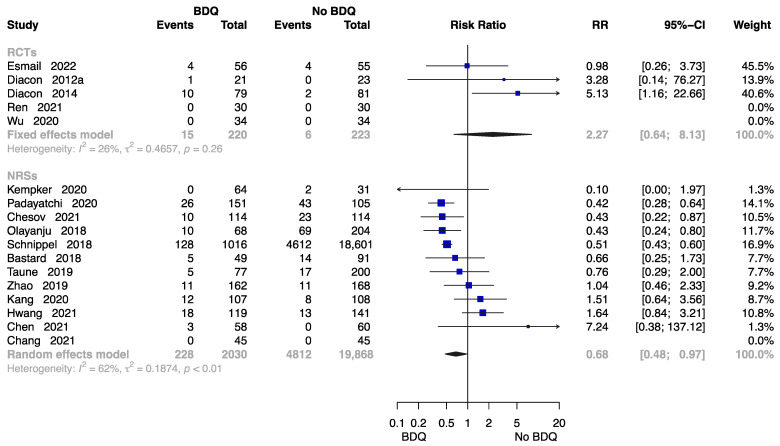
Forest plot of the rate of all-cause death at end of treatment. BDQ: bedaquiline; RCTs: randomized controlled trials; NRSs: non-randomized studies; RR: relative risks; CI: confidence interval. If heterogeneity *I*^2^ < 50% and *p*-value > 0.01, we used a fixed effects model; if heterogeneity *I*^2^ > 50% or *p*-value < 0.01, we used a random effects model [11,13,15,18,21,23,24,26,27,28,29,30,32,34,35,37,38].

Seventeen studies reported the rate of all-cause death at end of the treatment [11,13,15,18,21,23,24,26,27,28,29,30,32,34,35,37,38], with data available on 22,341 participants. Results from five RCTs and twelve NRSs were inconsistent. The RCTs showed no significantly higher risk of all-cause death in those who initiated a BDQ-containing regimen (RR = 2.27, M-H random-effects 95% CI: 0.64 to 8.13). However, the findings suggested the BDQ-containing regimen had a significantly lower risk of all-cause death (RR = 0.68, M-H random-effects 95% CI: 0.48 to 0.97) in NRSs (Figure 6).

There was a general symmetry to the funnel plot for estimating publication bias for NRSs (Figure S5), and Egger’s test found no publication bias (*p* = 0.163). No funnel plot of RCTs has been included as there were fewer than 10 studies.

##### The Failure Rate at End of the Treatment

**Figure 7:**
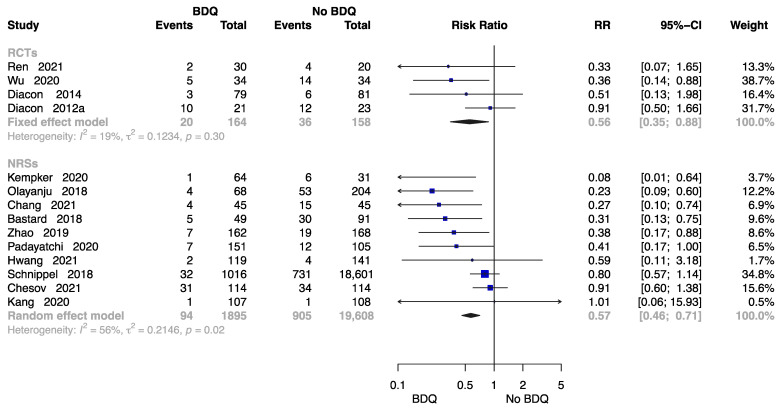
Forest plot of the rate of failure at end of treatment. BDQ: bedaquiline; RCTs: randomized controlled trials; NRSs: non-randomized studies; RR: relative risks; CI: confidence interval. If heterogeneity *I*^2^ < 50% and *p*-value > 0.01, we used a fixed effects model; if heterogeneity *I*^2^ > 50% or *p*-value < 0.01, we used a random effects model [11,13,18,21,23,24,26,28,29,30,32,34,37,38].

Fourteen studies were available to analyse the rate of failure at end of the treatment [11,13,18,21,23,24,26,28,29,30,32,34,37,38] including 2059 individuals administered a BDQ-containing regimen and 19,766 individuals with the no-BDQ-containing treatment, four of which were RCTs. The results revealed that the BDQ-containing regimen reduced the risk of failure rate significantly both in RCTs (RR = 0.56, 95% CI: 0.56 to 0.88) and in NRSs (RR = 0.57, 95% CI: 0.46 to 0.71) compared with the no-BDQ-containing regimen (Figure 7).

According to the assessment of publication bias, Egger’s test showed asymmetry for the outcomes of failure rate at end of the treatment in NRSs (*p* = 0.017). Using the trim-and-fill method, the adjust *p*-value was 0.907 (Figure S6).

##### The Rate of Lost to Follow-Up at End of the Treatment

Fourteen studies [11,13,15,23,24,26,28,29,30,32,34,35,37,38] collected data to assess the rate of lost to follow-up with 22,096 participants, three of which were RCTs. The forest plot shows an RR of 1.99 (M-H random-effects 95% CI: 0.61–6.51) in RCTs, and an RR of 0.84 (M-H random-effects 95% CI: 0.58–1.21) in NRSs. No statistically significant difference was detected in the lost to follow-up rate between the intervention and control groups (Figure S7).

In NRSs, Egger’s test indicated asymmetry for the lost to follow-up rate (*p* = 0.044) at the end of treatment based on an assessment of publication bias. The trim-and-fill method showed an adjust *p*-value of 0.698 (Figure S8).

### 2.4. Safety of Interventions

#### 2.4.1. Cardiotoxicity

**Figure 8:**
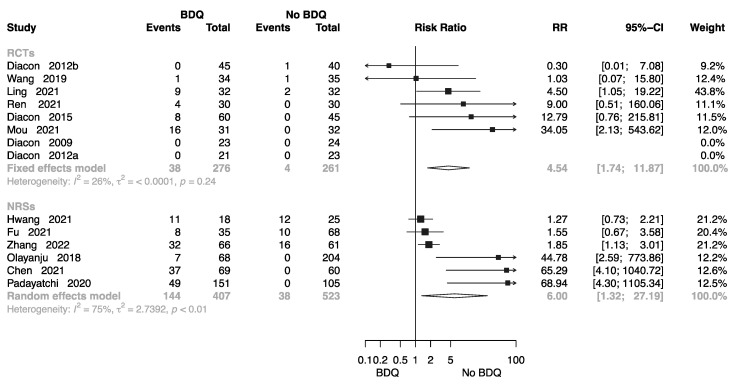
Forest plot of cardiotoxicity in studies of tuberculosis patients receiving BDQ. BDQ: bedaquiline; RCTs: randomized controlled trials; NRSs: non-randomized studies; RR: relative risks; CI: confidence interval. If heterogeneity *I*^2^ < 50% and *p*-value > 0.01, we used a fixed effects model; if heterogeneity *I*^2^ > 50% or *p*-value < 0.01, we used a random effects model [10,11,12,14,16,17,18,20,22,26,27,34,36,38].

Eight RCTs involving 537 individuals provided data for calculation of electrocardiogram (ECG) findings [10,11,12,14,16,17,18,20,22,26,27,32,36,38]; the pooled RR was 4.54 (M-H random-effects 95% CI: 1.74–11.87). Six NRSs involving 930 individuals provided data or calculation of ECG findings; the pooled RR was 6.00 (M-H random-effects 95% CI: 1.32–27.19). Overall, BDQ-containing regimens had a greater risk of cardiotoxicity than those that did not (Figure 8).

#### 2.4.2. Hepatotoxicity

Thirteen studies provided data on the comparison between BDQ-containing regimens and no-BDQ-containing regimens among TB patients [12,16,17,19,20,21,22,23,24,26,27,32,36]. The pooled results of RCTs (RR:1.61, 95% CI: 0.92–2.85) and NRSs (RR:1.10, 95% CI: 0.55–2.23) showed no significant difference in the incidence of hepatotoxicity (Figure S9).

#### 2.4.3. Grade 3–5 Adverse Events

**Figure 9:**
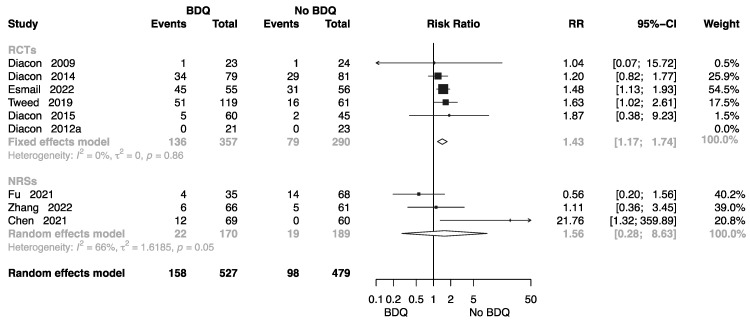
Forest plot of incidence of grade 3–5 adverse events in studies of tuberculosis patients receiving bedaquiline. BDQ: bedaquiline; RCTs: randomized controlled trials; NRSs: non-randomized studies; RR: relative risks; CI: confidence interval. If heterogeneity *I*^2^ < 50% and *p*-value > 0.01, we used a fixed effects model; if heterogeneity *I*^2^ > 50% or *p*-value < 0.01, we used a random effects model [10,11,13,14,15,19,22,27,36].

Eight studies collected related data to assess the incidence of grade 3–5 adverse events [10,11,13,14,15,19,22,27,36] in different regimen groups, five of which were RCTs. The forest plot shows an RR of 1.43 (M-H random-effects 95% CI: 1.17–1.74) in RCTs, and an RR of 1.56 (M-H random-effects 95% CI: 0.28–8.63) in NRSs (Figure 9).

### 2.5. Quality of Evidence Assessment

Quality of evidence were rated on a comparison between BDQ-containing and no-BDQ-containing regimen among patients with TB. Certainty of evidence ranges from very low to moderate for the RCTs and from very low to moderate for the NRSs (Table S2).

### 2.6. Subgroup Analysis

Subgroup analysis was conducted by splitting the “control group” into “background (BR) + no other treatment”, “BR + placebo”, “BR + second-line injectable drug (SLID)”, “BR + standard-of-care (SOC)”, and “BR + delamanid (DLM)” separately. Results of the rate of all-death cause death at end of treatment from RCTs and NRSs were inconsistent. The RCTs showed a significantly higher risk in those who initiated BDQ compared to placebo (RR = 4.73, 95% CI: 1.23–18.12), while the results from NRSs suggested a significantly lower risk (BR + BDQ vs. BR + no other treatment: RR = 0.59, 95% CI: 0.40–0.87; BR + BDQ vs. BR + SLID: RR = 0.51, 95% CI: 0.43–0.60; BR + BDQ vs. BR + DLM: RR = 0.85, 95% CI: 0.48–0.97) (Table S3).

### 2.7. Sensitivity Analysis

Sensitivity analysis was performed with the leave-one-out method in RCTs (Figures S10–S17). The pooled RRs remained consistent across the meta-analysis of studies, indicating that the results were robust except for three studies. Esmail 2022 [15], Wu 2020 [21], and Ling 2021 [16] showed inconsistency in the sensitivity analysis. When Esmail 2022 [15] was excluded, the RR changed from 2.27 (95% CI: 0.64–8.13) to 4.73 (95% CI: 1.23–18.12) in the all-cause death rate (Figure S9), and the RR changed from 1.39 (95% CI: 1.12–1.72) to 1.23 (95% CI: 0.85–1.78) in the incidence of grade 3–5 adverse events (Figure S13). As a result of Wu 2020 [21] being excluded, the pooled RR in the failure rate increased from 0.56 (95% CI: 0.35–0.88) to 0.68 (95% CI: 0.40–1.16) (Figure S10). When the study by Ling 2021 [16] was excluded, the pooled RR changed from 4.54 (95% CI: 1.74–11.87) to 4.14 (95% CI: 0.84–23.12) in the incidence of cardiotoxicity (Figure S12). These studies focused on the adverse effects of the administration of BDQ.

## 3. Materials and Methods

### 3.1. Protocol Registration

The Preferred Reporting Items for Systematic Reviews and Meta-Analyses (PRISMA) [42] and the Meta-analysis of Observational Studies in Epidemiology (MOOSE) were used to complete the report [43]. The study protocol was registered (registered number: CRD42022347057, www.crd.york.ac.uk/prospero/ (accessed on 28 August 2023)) with the International Prospective Register of Systematic Reviews (PROSPERO) following the standard reporting method [44].

### 3.2. Data Sources

The databases of MEDLINE (PubMed), the Web of Science, the Elton B. Stephens Company (EBSCO), the Cochrane Central Register of Controlled Trials, and the China National Knowledge Infrastructure (CNKI) were systematically searched to identify relevant trials. The search terms used included “Bedaquiline”, “BDQ”, “TMC207”, and “R207910”. No language restrictions were applied during the search process. The literature search was regularly updated on a weekly basis. The search was conducted from the inception of each database, with the initial search taking place on 15 June 2022, and updated to include the latest research on 20 March 2023.

### 3.3. Selection Criteria for Trials

Eligibility criteria for the included trials were as follows:

(1) Study population: Adults (>18 years old) with a clinical diagnosis of pulmonary tuberculosis (PTB), regardless of HIV status [45,46].
(2) Intervention: Anti-TB treatment (ATT) regimen with BDQ or BDQ-containing regimens at any dose and for any duration.
(3) Comparator: Another ATT regimen without BDQ.
(4) Primary outcomes: Efficacy of BDQ-containing treatment regimen, including the rate of sputum culture conversion, cure, all-cause death, treatment failure, and lost to follow-up at the end of treatment.
(5) Secondary outcome: Safety of BDQ-containing treatment regimen, including the incidence of cardiotoxicity, hepatotoxicity, and grade 3–5 adverse events during the treatment period.
(6) Study design: Included RCTs and NRSs such as retrospective cohort studies (RCSs) or prospective cohort studies (PCSs).
(7) Exclusion: Studies without a comparison group were excluded from the analysis.

### 3.4. Trial Identification

Two investigators independently screened articles by title, abstract, and full text using the inclusion criteria. The inclusion of a study was decided by consensus between the two investigators. When disagreements occurred, investigators consulted or discussed with a third one to solve them.

### 3.5. Data Extraction

The following study characteristics were extracted for eligible studies: (1) trail information: the first author, study year, study country; (2) patient characteristics: baseline characteristics of participants, time of recruitment, sample size per each treatment, regimens and arms used, and duration on treatment.

### 3.6. Quality and Risk of Bias Assessment

The quality of each eligible trial was assessed independently by two investigators based on the Cochrane risk of bias 2.0 (ROB 2.0) tool in RCTs [47] and the Risk of Bias in Non-randomized Studies of Interventions (ROBINS-I) tool for NRSs [48]. Data from different RCTs and NRSs was not pooled due to inconsistency in the direction of effect. Chi-square test with a *p* value of 0.10 were used to indicate statistical significance [49], and the *I*^2^ value of 50% was taken to indicate significant statistical heterogeneity [50]. Forest plots were used to visually assess the included trials.

### 3.7. Definitions

The definitions of treatment outcomes were based completely on those specified by the WHO for TB program outcomes [51]. We followed these guidelines to define and categorize the treatment outcomes in our study. The specific definitions and criteria for each outcome were extracted and documented. The number of participants who experienced each outcome was also recorded and summarized in Table S1.

### 3.8. Data Synthesis and Analysis

Dichotomous results are presented as relative risks (RR) and 95% confidence intervals (Cl). A random-effect model was used to conduct this meta-analysis when the significant variability in the sample of participants from different studies, and a fixed-effect model was used when heterogeneity was low (*I*^2^ < 50% and *p* > 0.01) [50].

Dichotomous results were analysed using relative risks (RR) and 95% confidence intervals (CI). A random-effects model was employed for the meta-analysis in the presence of significant variability among the study participants from different studies. Conversely, a fixed-effect model was used when heterogeneity was low, as indicated by an *I*^2^ value below 50% and a *p*-value above 0.01.

The Mantel–Haenszel method was applied without continuity correction when at least one study reported zero events in one group [52]. Missing sample values were calculated from the description in the *Cochrane Handbook* [53].

To assess publication bias, funnel plots and Egger’s test were utilized when there were at least 10 studies included. Any substantial asymmetry observed in the funnel plots could indicate the presence of publication bias. In such cases, adjustment for potential publication bias was performed using the trim-and-fill method [54]. The analysis was planned to be repeated by conducting subgroup analyses for different interventions in the control group and by excluding data derived from imputed methods. Sensitivity analyses using the leave-one-out method were performed specifically for the randomized controlled trials (RCTs).

All analyses were conducted using the meta package version 5.2-0 of R Version 4.1.2 (RStudio, Boston, MA, USA) [55,56]. The Grading of recommendations assessment, development, and evaluation (GRADE) approach was used to assess the certainty of the evidence by GRADEpro GDT software (McMaster University and Evidence Prime 2022. Available from gradepro.org) [57].

## 4. Discussion

### 4.1. Summary of Findings

BDQ, the first new TB drug to be approved in over 40 years, is a narrow-spectrum antibiotic primarily active against mycobacteria [58]. BDQ targets ATP synthase by inhibiting the proton pumping mechanism [3,59]. It is well documented that BDQ can bind to the oligomeric/proteolipidic subunit C in the F_0_ domain of the ATP synthase complex and prevent its function [60]. Several trials have reported that BDQ can reduce the time required for sputum smear and culture conversion in patients with MDR-TB [10,11]. However, BDQ has a black-box warning related to efficacy and safety [61].

According to a meta-analysis conducted by the WHO involving 391 patients with drug-resistant TB, 69% of patients treated with BDQ achieved successful outcomes, with nearly 80% showing sputum culture conversion after 24 weeks of treatment [62]. Importantly, our study demonstrated that the inclusion of BDQ in the treatment regimen resulted in improved rates of sputum conversion at 24 weeks and during follow-up, when compared to regimens without BDQ in RCTs. Furthermore, in NRSs, BDQ-containing regimens were associated with improved sputum culture conversion rates during follow-up. These findings suggest that the use of BDQ may lead to better treatment outcomes, including shorter MDR-TB regimens and improved sputum culture conversion rates during follow-up. However, it is crucial to evaluate these findings in RCTs that specifically assess the early term rates of sputum culture conversion.

Data from five cohorts conducted in the USA, France, South Africa, Georgia, and Armenia were analysed to evaluate the treatment outcomes of BDQ. The reported results indicated a cure rate of 63.8%, a treatment success rate of 69.1%, and an all-cause death rate of 10.6% [63]. In our study, we found the BDQ-containing regimen increased the cure rate and decreased failure rate, and there was not differ significantly in the rate of all-cause death in RCTs, the subgroup analysis revealed a significantly higher all-cause death rate with BDQ use compared with a placebo. It is important to note that BDQ exhibits extensive tissue distribution with intracellular accumulation. The time to peak concentration and plasma half-life of BDQ are approximately 4–6 h and 24–30 h, respectively, with an exceptionally long terminal elimination half-life [58,60]. Future studies will need to determine the impact of these pharmacokinetic characteristics on toxic effects and mortality.

The frequency of ventricular arrhythmia exhibits a rise of 5 to 7% for each 10 ms elevation in QTc value [64]. It has been observed in TB patients that an increased QTc interval on the ECG is one of the most common side effects of BDQ [6]. Borisov’s multicentre study illustrated that, among patients grappling with MDR-TB and XDR-TB, the inclusion of BDQ in an optimally structured regimen led to a QTcF prolongation exceeding 500 ms in 9.7% (24 out of 247) of cases [65]. Gao’s research, which encompassed a cohort of 177 patients, illustrates that the primary rationale for discontinuation of BDQ was the elongation of the QTc interval, specifically, 10 individuals (5.6%) exhibited QTc prolongation equal to or surpassing 500 ms [66]. Similarly, in another substantial study conducted by TBNet, involving a cohort of 1044 individuals receiving BDQ for tuberculosis, the cessation of the drug occurred in eight cases owing to QTcF prolongation (0.77%, 95% CI: 0.04–1.57%) [67]. In our study, we found that the incidence of cardiotoxicity and grade 3-5 adverse events was more prevalent in the randomized controlled trials (RCTs). Additionally, the administration of BDQ-containing regimens was associated with cardiotoxicity in the non-randomized studies (NRSs). Therefore, it is crucial to carefully and regularly monitor patients for serious side effects during BDQ administration and even after discontinuation of the drug. Prospective trials ought to accord paramount importance to the vigilant monitoring of safety aspects, given their substantive implications within clinical and public health paradigms.

In this systematic review and meta-analysis, we aimed to summarize the efficacy- and safety-related outcomes of TB patients treated with BDQ-containing regimens. However, we observed inconsistent associations between the BDQ-containing regimen and efficacy- and safety-related outcomes across different studies. Several potential reasons may account for this discrepancy. Firstly, differences in participants’ characteristics and settings at baseline may play a role. While all participants in the RCTs were from countries burdened by high rates of TB, TB/HIV coinfection, and MDR-TB, some of the NRSs included trials conducted in countries with lower TB incidence. This variation suggests that participants in the RCTs may have been at a higher risk of TB compared to those in the NRSs. Secondly, the therapeutic effect of TB is not solely determined by the drug regimen but is also influenced by environmental factors, concomitant diseases, immunosuppressive drugs, and the immunological status of the host [68]. Unfortunately, some of the studies we included did not provide detailed information on these important factors.

Our findings differ from a previous review conducted by Hossein et al. [69], which focused on data from observational studies and experimental studies. They reported a pooled treatment success rate of 74.7% for BDQ-containing regimens in observational studies and 86.1% in experimental studies. Another meta-analysis conducted by Wang [70] indicated that BDQ-containing regimens were associated with improved rates of culture conversion (RR = 1.27, 95% CI: 1.17–1.39; n = 6 studies) and reduced all-cause death (RR = 0.53, 95% CI: 0.45–0.62; n=6 studies) compared to the control group. In contrast, our analysis did not find a significant association between BDQ-containing regimens and the rate of all-cause death. This discrepancy may be attributed to the inclusion of a larger number of relevant studies in our meta-analysis and the separate pooling of data from RCTs and NRSs, resulting in a larger dataset for analysis. RCTs are known for their strong internal validity and are considered a superior study design [71]. Therefore, future analyses should aim to include more randomized controlled trials to further confirm our findings. 

The novel therapeutic options introduced by WHO regarding BDQ represent a groundbreaking advancement in the treatment of MDR/RR-TB. This development heralds a new era in the management of this challenging disease, offering an all-oral, shorter, and more patient-centred treatment approach. It presents a promising model for MDR/RR-TB management that is not only more acceptable to patients but also more equitable and cost-effective [72,73]. Questions remain about optimizing the duration and combination of bedaquiline efficacy and safety in multidrug-resistant/RR-TB therapy. In future programs, it is imperative to adhere rigorously to TB drug monitoring initiatives, considering each patient’s individual situation, and conducting comprehensive assessments to weigh the benefits and risks of the chosen treatment regimen. This entails meticulous attention to detail in the utilization of digital technology, ensuring accurate recording and reporting of post-treatment follow-up data to evaluate treatment success and the risk of relapse. Moreover, particular attention should be given to the tolerability and safety of the regimen to optimize treatment outcomes and enhance patient well-being. By implementing these strategies, we can achieve more effective and successful management of MDR/RR-TB cases, significantly impacting public health efforts to combat tuberculosis.

### 4.2. Strengths and Limitations

This review has some strengths. To our knowledge, this is the relatively comprehensive systematic review of prospective and retrospective cohort studies and RCTs relevant to the assessment of the efficacy and safety of BDQ in the treatment of tuberculosis. The studies recruited participants from high- and low- TB-, TB/HIV-, and MDR-TB-burden countries, and therefore are representative across a wide range of TB program settings.

This study has several limitations. First, the results of a meta-analysis are highly dependent on the quality of studies included. However, we included studies that used a non-randomized controlled trial design [22,23,24,25,26,27,28,29,30,31,32,33,34,35,36,37,38], which was more susceptible to biases, and according to the GRADE results, the assessments of the certainty of the evidence ranged from moderate to very low, and there was some uncertainty regarding all the estimates. Second, it is difficult to standardize methods in these types of studies, due to the different background treatment regimens that are possible, the constant updating of medication protocols, and the complexity of changes in patient behaviours; thus, variability among different studies influences the results of our meta-analysis. Third, small trials tend to report larger beneficial effects than large trials [74], and our review reports only five trials including more than 100 patients per arm, which may introduce bias due to small study effects. Fourth, our review combined different dosages and accompanying regimens into one group, and the control group also included several treatments of a no-BDQ-containing regimen; these would not have clarified the individual contribution of BDQ. Fifth, our review only included English and Chinese language articles; journals in other languages and other electronic databases are lacking potentially relevant studies, which might have influenced the pooled estimated value. Considering these limitations, our findings should be interpreted with caution.

## 5. Conclusions

In conclusion, this systematic review and meta-analysis provide evidence regarding the outcomes of BDQ-containing regimens compared to regimens without BDQ for patients with TB. The findings suggest that the use of BDQ in TB treatment is both feasible and effective. BDQ-containing regimens demonstrated a higher rate of sputum culture conversion and a higher rate of cure compared to regimens without BDQ. Additionally, the risk of treatment failure was found to be lower in the BDQ-containing group. However, it is important to note that the use of BDQ was associated with an increased incidence of cardiotoxicity.

To obtain more precise and robust results, large-scale multicentre clinical trials are warranted. These trials would contribute to a better understanding of the efficacy and safety profile of BDQ in the treatment of TB.

## Data Availability

Dataset is available from the corresponding author after reasonable request.

## Supplementary Materials

The following supporting information can be downloaded at: https://www.mdpi.com/article/10.3390/antibiotics12091389/s1, Table S1: Definition of treatment outcomes; Table S2: Grading of recommendations assessment, development, and evaluation (GRADE) methodology in assessment of evidence regarding bedaquiline-containing regimen compared no bedaquiline-containing regimen for patients with TB; Table S3: summary of results for the subgroup analyses, provided that excluded data for the imputed methods; Figure S1: Risk of Bias, showing the domain assessment for individual trials; Figure S2: Forest plot of the rate of sputum culture conversion at 8 weeks; Figure S3: Funnel plot of NRSs comparing culture conversion rates at 24 weeks in TB patients undergoing bedaquiline-containing regimen versus no-bedaquiline-containing regimen; Figure S4: Forest plot of the rate of complete at end of treatment; Figure S5: Funnel plot of NRSs comparing all-cause death at end of the treatment in TB patients undergoing bedaquiline-containing regimen versus no bedaquiline-containing regimen; Figure S6: Funnel plot of NRSs comparing failure rate at end of the treatment in TB patients undergoing bedaquiline-containing regimen versus no-bedaquiline-containing regimen; Figure S7: Forest plot of the rate of lost to follow-up at end of treatment; Figure S8: Funnel plot of NRSs comparing the rate of lost to follow-up at the end of treatment at end of the treatment in TB patients undergoing bedaquiline-containing regimen versus no bedaquiline-containing regimen; Figure S9: Forest plot of incidence of hepatotoxicity in studies of tuberculosis patients receiving bedaquiline; Figure S10: Sensitivity analysis of the rate of sputum culture conversion at 8 weeks in RCTs; Figure S11: Sensitivity analysis of the rate of sputum culture conversion at 24 weeks in RCTs; Figure S12: Sensitivity analysis of the rate of complete at end of the treatment in RCTs; Figure S13: Sensitivity analysis of the rate of all-cause death at end of the treatment in RCTs; Figure S14: Sensitivity analysis of the failure rate at end of the treatment in RCTs; Figure S15: Sensitivity analysis of the incidence of cardiotoxicity in RCTs; Figure S16: Sensitivity analysis of the incidence of hepatotoxicity in RCTs; Figure S17: Sensitivity analysis of the incidence of grade 3–5 adverse events in RCTs.
