# Supplementary material for: The Efficacy and Safety of Bedaquiline in the Treatment of Pulmonary Tuberculosis Patients: A Systematic Review and Meta-Analysis
Source: Antibiotics (Basel). 2023 Aug 31;12(9):1389. doi: 10.3390/antibiotics12091389 (PMC10525131; doi:10.3390/antibiotics12091389)
Supplement: Supplementary file 1 [file antibiotics-12-01389-s001.zip › antibiotics-2528176-supplementary-updated.pdf]

Supplementary material：

Supplementary Tables:

Table S1. Definition of treatment outcomes

Table S2. Grading of recommendations assessment, development, and evaluation (GRADE) methodology in assessment of evidence regarding bedaquiline-containing regimen compared no bedaquiline-containing regimen for patients with TB

Table S3. summary of results for the subgroup analyses, provided that excluded data for the imputed methods.

Supplementary Figures:

Figure S1. Risk of Bias, showing the domain assessment for individual trials

Figure S2. Forest plot of the rate of sputum culture conversion at 8 weeks.

Figure S3. Funnel plot of NRSs comparing culture conversion rates at 24 weeks in TB patients undergoing bedaquiline-containing regimen versus no bedaquiline-containing regimen.

Figure S4. Forest plot of the rate of complete at end of treatment

Figure S5 Funnel plot of NRSs comparing all-cause death at end of the treatment in TB patients undergoing bedaquiline-containing regimen versus no bedaquiline-containing regimen.

Figure S6 Funnel plot of NRSs comparing failure rate at end of the treatment in TB patients undergoing bedaquiline-containing regimen versus no bedaquiline-containing regimen.

Figure S7. Forest plot of the rate of lost to follow-up at end of treatment

Figure S8. Funnel plot of NRSs comparing the rate of lost to follow-up at the end of treatment at end of the treatment in TB patients undergoing bedaquiline-containing regimen versus no bedaquiline-containing regimen.

Figure S9. Forest plot of incidence of hepatotoxicity in studies tuberculosis patients receiving bedaquiline

Figure S10. Sensitivity analysis of the rate of sputum culture conversion at 8 weeks in RCTs

Figure S11. Sensitivity analysis of the rate of sputum culture conversion at 24 weeks in RCTs

Figure S12. Sensitivity analysis of the rate of complete at end of the treatment in RCTs

Figure S13. Sensitivity analysis of the rate of all-cause death at end of the treatment in RCTs

Figure S14. Sensitivity analysis of the failure rate at end of the treatment in RCTs

Figure S15. Sensitivity analysis of the incidence of cardiotoxicity in RCTs

Figure S16. Sensitivity analysis of the incidence of hepatotoxicity in RCTs

Figure S17. Sensitivity analysis of the incidence of grade 3-5 adverse events in RCTs

Table S1 Definition of treatment outcomes

Treatment outcome	definition
Sputum culture conversion	culture is considered to have converted to negative when two consecutive cultures, taken at least 30 days apart, are found to be negative.
Treatment completion	Treatment completed as recommended by the national policy without evidence of failure but no record that three or more consecutive cultures taken at least 30 days apart are negative after the intensive phase.
Cure	Treatment completed as recommended by the national policy without evidence of failure and three or more consecutive cultures taken at least 30 days apart are negative after the intensive phase.
All-cause death	A patient who dies for any reason during the course of treatment.
Treatment failure	Treatment terminated or need for permanent regimen change of at least two anti-TB drugs because of:<br>– lack of conversion by the end of the intensive phase , or<br>– bacteriological reversion in the continuation phase after conversion to negative, or<br>– evidence of additional acquired resistance to fluoroquinolones or second-line injectable drugs, or<br>– adverse drug reactions (ADRs).
Lost to follow-up	A patient whose treatment was interrupted for 2 consecutive months or more.

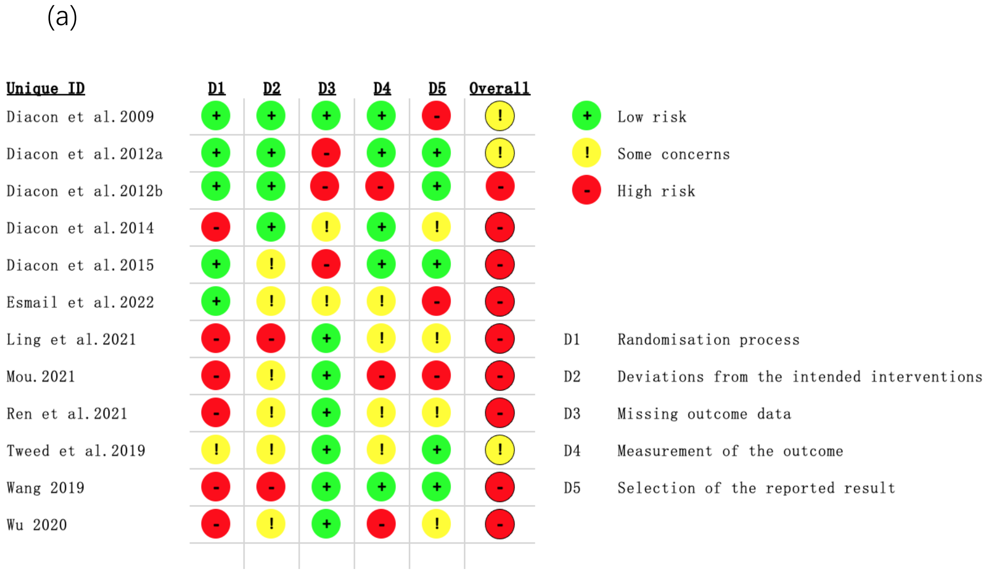

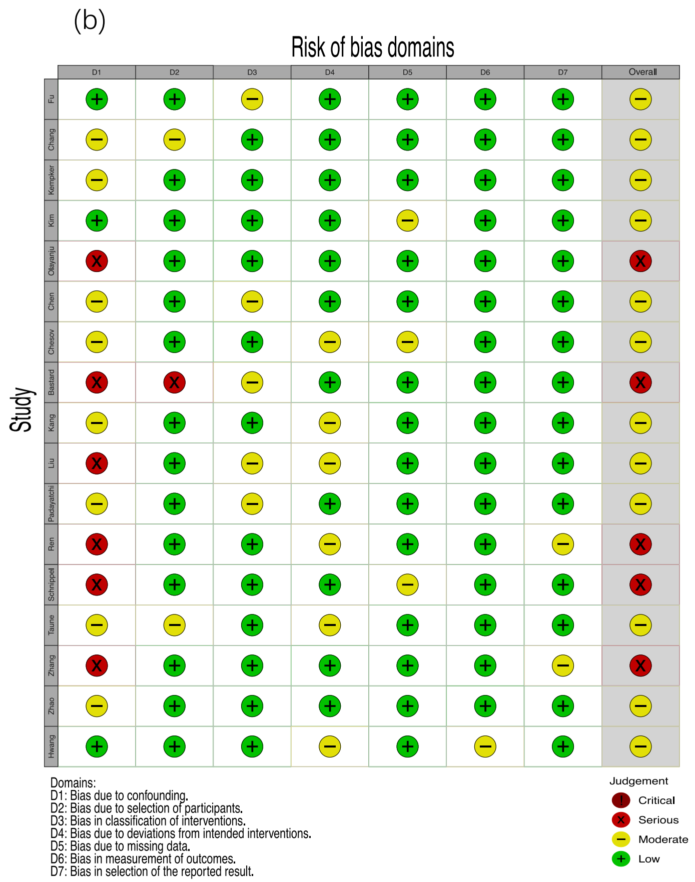

Figure S1. Ri sk of Bias, showing the domain assessment for individual trials. (a) randomised controlled trials. (b) non-randomised controlled trials.

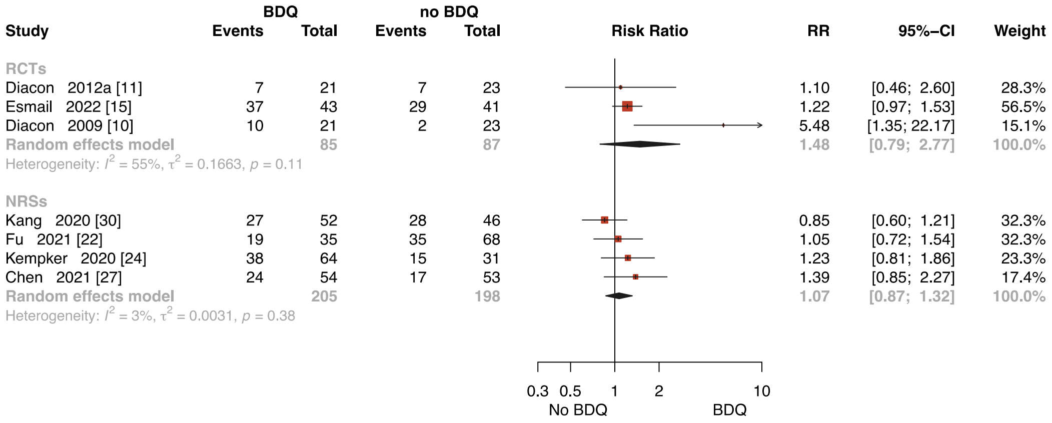

Figure S2. Forest plot of the rate of sputum culture conversion at 8 weeks.

BDQ: bedaquiline; RCTs: randomized controlled trials; NRSs: non-randomized studies; RR: relative risks CI: confidence interval. If heterogeneity $I^2 < 50\%$ and p-value>0.01, we used a fixed effects model; if heterogeneity $I^2 > 50\%$ or p-value <0.01, we used a random effects model [10,11,15,22,24,27,30].

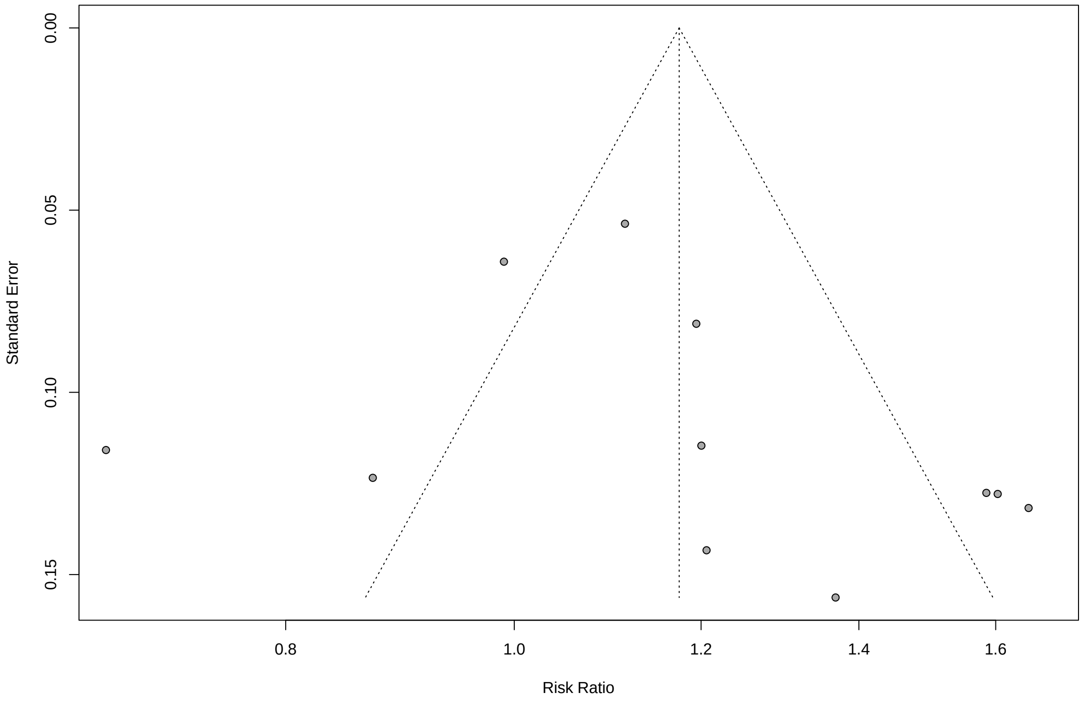

Figure S3. Funnel plot of NRSs comparing culture conversion rates at 24 weeks in TB patients undergoing bedaquiline-containing regimen versus no bedaquiline-containing regimen. No funnel plot of RCTs has been included as there were fewer than 10 studies. NRS: non-randomised study; RCT: randomised controlled trial; TB: tuberculosis

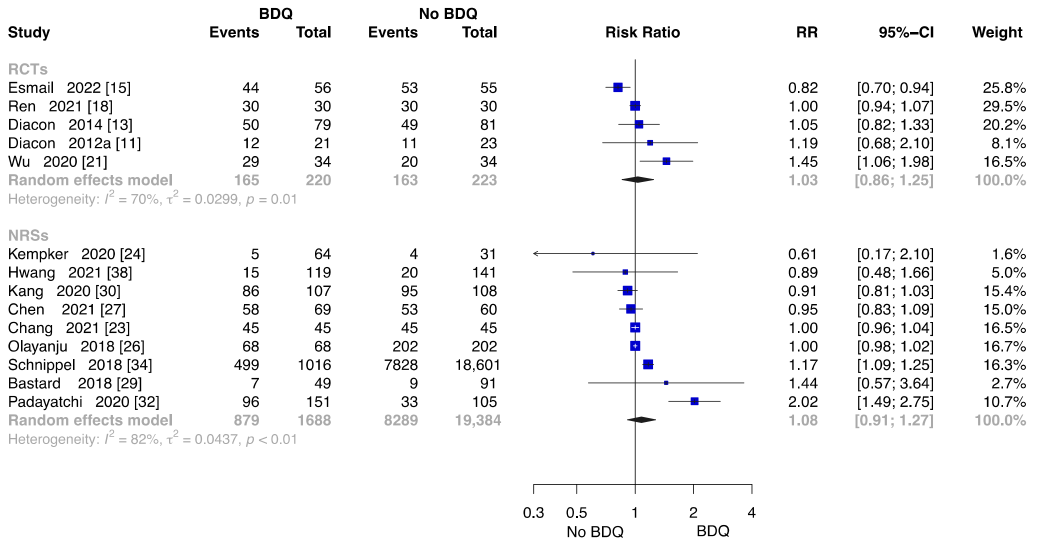

Figure S4. Forest plot of the rate of complete at end of treatment

BDQ: bedaquiline; RCTs: randomized controlled trials; NRSs: non-randomized studies; RR: relative risks CI: confidence interval. If heterogeneity $I^2 < 50\%$ and p-value>0.01, we used a fixed effects model; if heterogeneity $I^2 > 50\%$ or p-value <0.01, we used a random effects model [11,13,15,18,21,23-24,26,27,29,30,32,34,38].

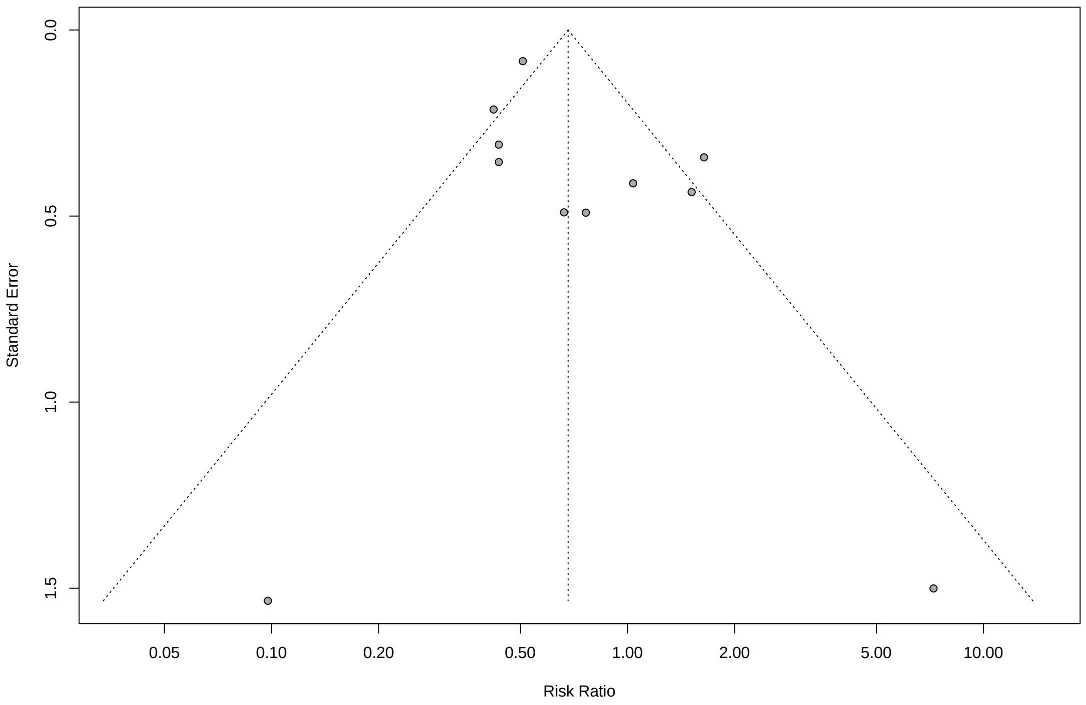

Figure S5 Funnel plot of NRSs comparing all-cause death at end of the treatment in TB patients undergoing bedaquiline-containing regimen versus no bedaquiline-containing regimen. No funnel plot of RCTs has been included as there were fewer than 10 studies. NRS: non-randomised study;

RCT: randomised controlled trial; TB: tuberculosis

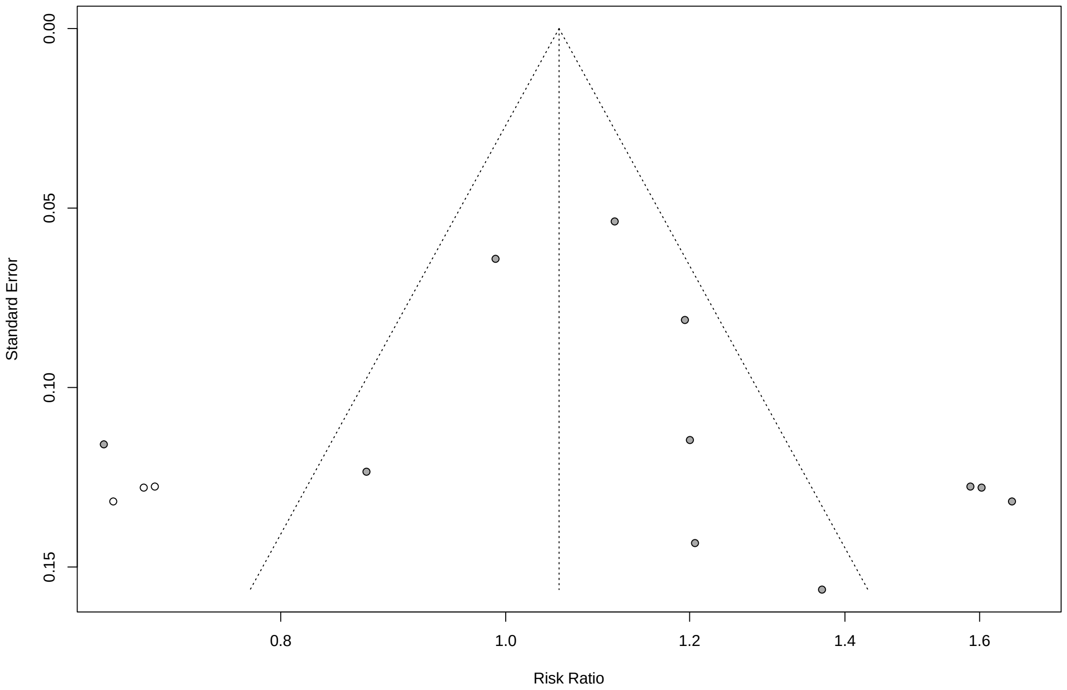

Figure S6 Funnel plot of NRSs comparing failure rate at end of the treatment in TB patients undergoing bedaquiline-containing regimen versus no bedaquiline-containing regimen. The solid circles represent the NRS units, and the empty circles represent studies trimmed. No funnel plot of RCTs has been included as there were fewer than 10 studies.

NRS: non-randomised study; RCT: randomised controlled trial; TB: tuberculosis.

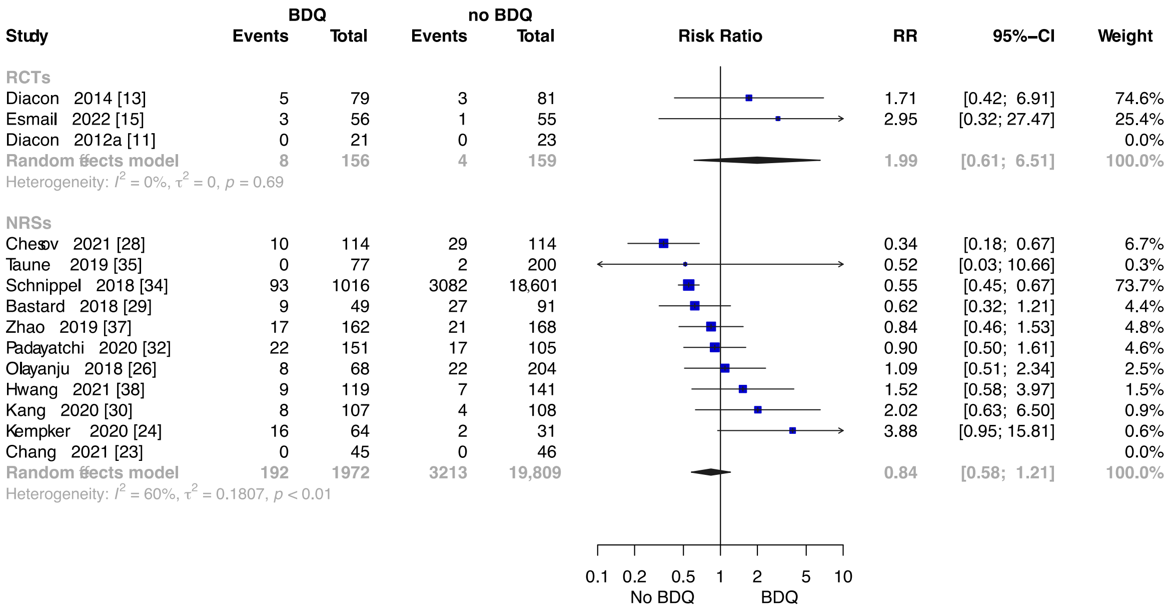

Figure S7 Forest plot of the rate of lost to follow-up at end of treatment. BDQ: bedaquiline; RCTs: randomized controlled trials; NRSs: non-randomized studies; RR: relative risks; CI: confidence interval. If heterogeneity I^2< 50% and p-value>0.01, we used a fixed effects model; if heterogeneity I^2>50% or p-value <0.01, we used a random effects model [11,13,15,23,24,26,28-30,32,34,35,37,38].

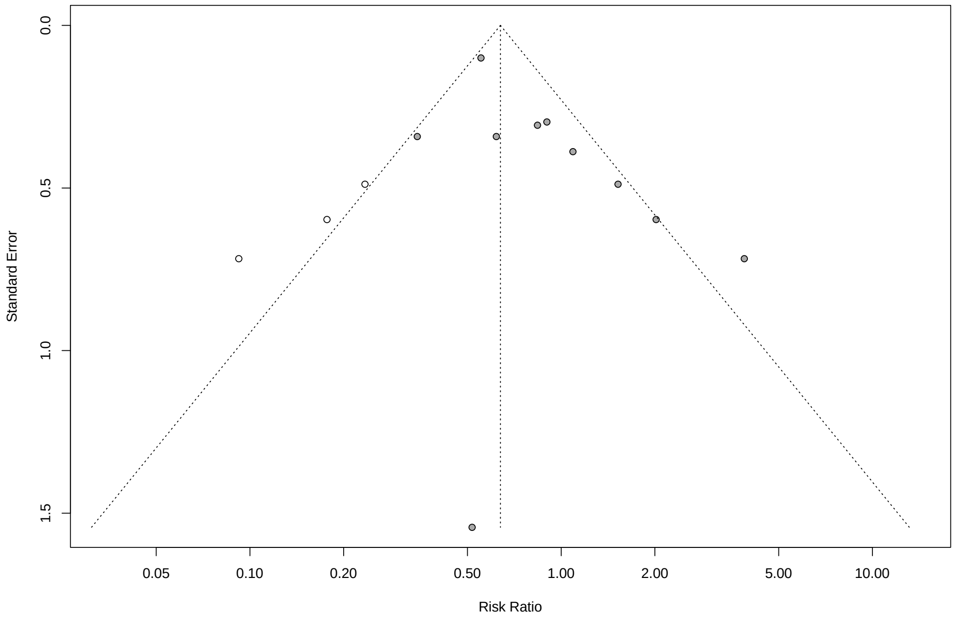

Figure S8. Funnel plot of NRSs comparing the rate of lost to follow-up at the end of treatment at end of the treatment in TB patients undergoing bedaquiline-containing regimen versus no bedaquiline-containing regimen. The solid circles represent the NRS units, and the empty circles represent studies trimmed. No funnel plot of RCTs has been included as there were fewer than 10 studies.

NRS: non-randomised study; RCT: randomised controlled trial; TB: tuberculosis

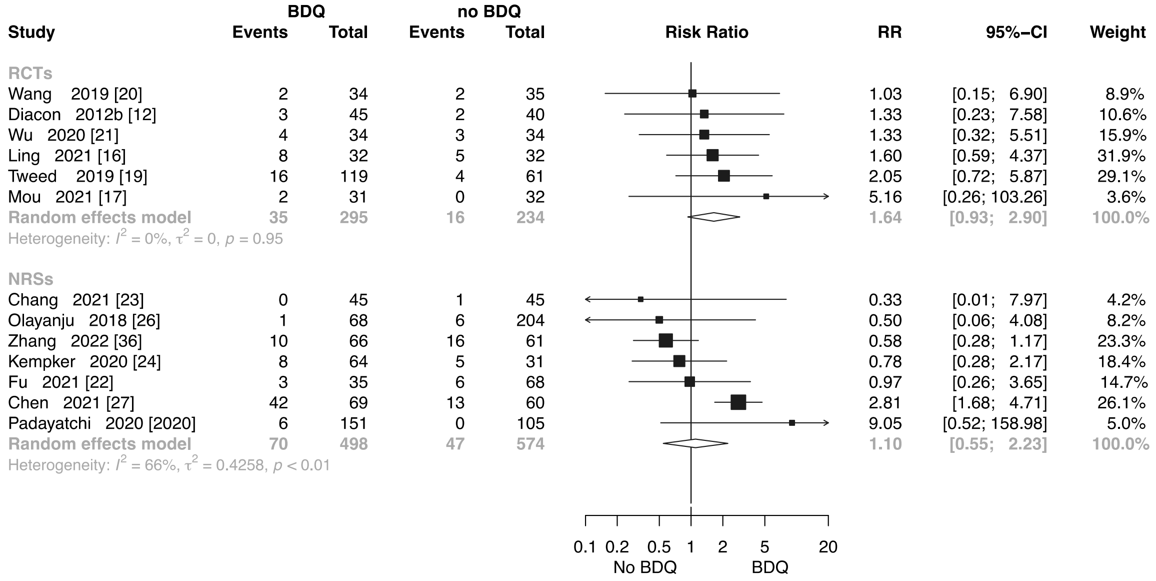

Figure S9. Forest plot of incidence of hepatotoxicity in studies tuberculosis patients receiving bedaquiline. BDQ: bedaquiline; RCTs: randomized controlled trials; NRSs: non-randomized studies; RR: relative risks CI: confidence interval. If heterogeneity I^2< 50% and p-value>0.01, we used a fixed effects model; if heterogeneity I^2>50% or p-value <0.01, we used a random effects model [12,16,17,19–24,26,27,32,36].

Table S2. Grading of recommendations assessment, development, and evaluation (GRADE) methodology in assessment of evidence regarding bedaquiline-containing regimen compared no bedaquiline-containing regimen for patients with TB.

Outcomes	studies	Number of patients		Effect		Certainty of the evidence (GRADE)
		BDQ-containing regimen	No BDQ-containing regimen	Relative (95%Cl)	Absolute (95%Cl)	
The rate of sputum culture conversion at 8 weeks	Two RCTs	54/85 (63.5%)	38/87 (43.7%)	RR 1.48 (0.79 to 2.77)	210 more per 1,000 (from 92 fewer to 773 more)	⊕⊕◯◯ Low
The rate of sputum culture conversion at 8 weeks	Four observational studies	108/205 (52.7%)	95/198 (48.0%)	RR 1.07 (0.87 to 1.32)	34 more per 1,000 (from 62 fewer to 154 more)	⊕⊕⊕◯ Moderate
The rate of sputum culture conversion at 24 weeks	Seven RCTs	241/257 (93.8%)	165/158 (104.4%)	RR 1.27 (1.10 to 1.46)	282 more per 1,000 (from 104 more to 480 more)	⊕⊕◯◯ Low
The rate of sputum culture conversion at 24 weeks	Eleven observational studies	576/770 (74.8%)	483/853 (56.6%)	RR 1.17 (1.00 to 1.38)	96 more per 1,000 (from 0 fewer to 215 more)	⊕◯◯◯ Very low
The rate of sputum culture conversion with follow-up	Two RCTs	63/87 (72.4%)	49/89 (55.1%)	RR 1.33 (1.06 to 1.66)	182 more per 1,000 (from 33 more to 363 more)	⊕⊕⊕◯ Moderate
The rate of culture conversion with follow-up	Three observational studies	643/1216 (52.9%)	7913/18937 (41.8%)	RR 1.53 (1.07 to 2.20)	221 more per 1,000 (from 29 more to 501 more)	⊕◯◯◯ Very low
The rate of complete at end of the treatment	Four RCTs	165/220 (75.0%)	163/223 (73.1%)	RR 1.03 (0.86 to 1.25)	22 more per 1,000 (from 102 fewer to 183 more)	⊕◯◯◯ Very low
The rate of complete at end of the treatment	Nine observational studies	879/1688 (52.1%)	8289/19384 (42.8%)	RR 1.08 (0.91 to 1.27)	34 more per 1,000 (from 38 fewer to 115 more)	⊕◯◯◯ Very low
The rate of cure at end of the treatment	Two RCTs	39/64 (60.9%)	24/64 (37.5%)	RR 1.60 (1.13 to 2.26)	225 more per 1,000 (from 49 more to 472 more)	⊕⊕⊕◯ Moderate

Table S2. Grading of recommendations assessment, development, and evaluation (GRADE) methodology in assessment of evidence regarding bedaquiline-containing regimen compared no bedaquiline-containing regimen for patients with TB (continued).

Outcomes	studies	Number of patients		Effect		Certainty of the evidence (GRADE)
		BDQ-containing regimen	No BDQ-containing regimen	Relative (95%Cl)	Absolute (95%Cl)	
The rate of cure at end of the treatment	Eight observational studies	883/1626 (54.3%)	8065/19332 (41.7%)	RR 1.86 (1.23 to 2.83)	359 more per 1,000 (from 96 more to 763 more)	⊕◯◯◯ Very low
The rate of all-cause death at end of the treatment	Five RCTs	15/220 (6.8%)	6/223 (2.7%)	RR 2.27 (0.64 to 8.13)	34 more per 1,000 (from 10 fewer to 192 more)	⊕⊕⊕◯ Moderate
The rate of all-cause death at end of the treatment	Twelve observational studies	228/2030 (11.2%)	4812/19868 (24.2%)	RR 0.68 (0.48 to 0.97)	78 fewer per 1,000 (from 126 fewer to 7 fewer)	⊕◯◯◯ Very low
The rate of failure at end of the treatment	Four RCTs	20/164 (12.2%)	36/158 (22.8%)	RR 0.56 (0.35 to 0.88)	100 fewer per 1,000 (from 148 fewer to 27 fewer)	⊕⊕⊕◯ Moderate
The rate of failure at end of the treatment	Ten observational studies	94/1895 (5.0%)	905/19608 (4.6%)	RR 0.57 (0.46 to 0.71)	20 fewer per 1,000 (from 25 fewer to 13 fewer)	⊕⊕◯◯ Low
The rate of lost to follow-up at end of the treatment	Three RCTs	8/156 (5.1%)	4/159 (2.5%)	RR 1.99 (0.61 to 6.51)	25 more per 1,000 (from 10 fewer to 139 more)	⊕⊕⊕◯ Moderate
The rate of lost to follow-up at end of the treatment	Eleven observational studies	192/1972 (9.7%)	3213/19809 (16.2%)	RR 0.84 (0.58 to 1.21)	26 fewer per 1,000 (from 68 fewer to 34 more)	⊕⊕⊕◯ Moderate
cardiotoxicity	Eight RCTs	38/276 (13.8%)	4/261 (1.5%)	RR 4.54 (1.74 to 11.87)	54 more per 1,000 (from 11 more to 167 more)	⊕⊕⊕◯ Moderate
cardiotoxicity	Six observational studies	144/407 (35.4%)	38/523 (7.3%)	RR 6.00 (1.32 to 27.19)	363 more per 1,000 (from 23 more to 1,000 more)	⊕⊕◯◯ Low

Table S2. Grading of recommendations assessment, development, and evaluation (GRADE) methodology in assessment of evidence regarding bedaquiline-containing regimen compared no bedaquiline-containing regimen for patients with TB (continued).

**Outcomes**	**studies**	**Number of patients**		**Effect**		**Certainty of the evidence (GRADE)**
		**BDQ-containing regimen**	**No BDQ-containing regimen**	**Relative (95%Cl)**	**Absolute (95%Cl)**	
Hepatotoxicity	Six RCTs	35/295 (11.9%)	16/234 (6.8%)	RR 1.64 (0.93 to 2.90)	44 more per 1,000 (from 5 fewer to 130 more)	⊕⊕◯◯ Low
Hepatotoxicity	Seven observational studies	70/498 (14.1%)	47/574 (8.2%)	RR 1.10 (0.55 to 2.23)	8 more per 1,000 (from 37 fewer to 101 more)	⊕◯◯◯ Very low
Grade 3-5 adverse events	Five RCTs	131/297 (44.1%)	77/245 (31.4%)	RR 1.42 (1.17 to 1.73)	132 more per 1,000 (from 53 more to 229 more)	⊕⊕◯◯ Low
Grade 3-5 adverse events	Three observational studies	22/170 (12.9%)	19/189 (10.1%)	RR 1.56 (0.28 to 8.63)	56 more per 1,000 (from 72 fewer to 767 more)	⊕⊕◯◯ Low

Table S3. summary of results for the subgroup analyses, provided that excluded data for the imputed methods (continued).

Outcomes		Type of intervention	RCT				NRS			
			**No. of studies**	**Result (95%Cl)**	I^2	P	**No. of studies**	**Result (95%Cl)**	I^2	P
The rate of culture conversion at 8 weeks	Main analyses		3	1.48(0.79,2.77)	55%	0.11	4	1.07(0.87,1.32)	3%	0.38
	Subgroup analyses	BR + BDQ VS BR + no other treatment	-	-	-	-	2	1.17(0.86-1.58)	0%	0.39
		BR + BDQ VS BR + placebo	2	2.22(0.97-1.53)	73%	0.06	-	-	-	-
		BR + BDQ VS BR +SOC	1	1.22(0.97-1.53)	-	-	-	-	-	-
		BR + BDQ VS BR + DLM	-	-	-	-	2	1.00(0.70-1.43)	42%	0.19
The rate of culture conversion at 24 weeks	Main analyses		7	**1.27(1.10-1.46)**	65%	<0.01	11	**1.17(1.00-1.38)**		
	Subgroup analyses	BR + BDQ VS BR + no other treatment	3	**1.27(1.17-1.68)**	0%	0.47	7	**1.30(1.09-1.54)**	76%	<0.01
		BR + BDQ VS BR + placebo	3	**1.32(1.13-1.55)**	0%	0.91	-	-	-	-
		BR + BDQ VS BR +SOC	1	**1.32(1.13-1.55)**	-	-	-	-	-	-
		BR + BDQ VS BR + DLM	-	-	-	-	4	0.99(0.76-1.28)	81%	<0.01
The rate of culture conversion with follow-up	Main analyses		2	**1.33(1.06-1.66)**	0%	0.49	3	**1.53(1.07-2.20)**	91%	<0.01
	Subgroup analyses	BR + BDQ VS BR + no other treatment	-	-	-	-	2	**1.81(1.26-2.61)**	68%	0.08
		BR + BDQ VS BR + placebo	2	**1.33(1.06-1.66)**	0%	0.49	-	-	-	-
		BR + BDQ VS BR +SOC	-	-	-	-	-	-	-	-
		BR + BDQ VS BR +SLID	-	-	-	-	1	**1.17(1.10-1.25)**	-	-
		BR + BDQ VS BR + DLM	-	-	-	-	-	-	-	-

Table S3. summary of results for the subgroup analyses, provided that excluded data for the imputed methods (continued).

**Outcomes**		**Type of intervention**	**RCT**				**NRS**			
			**No. of studies**	**Result (95%Cl)**	I^2	***P***	**No. of studies**	**Result (95%Cl)**	I^2	***P***
The rate of complete at end of treatment	Main analyses		5	1.03(0.86-1.25)	70%	0.01	9	1.08(0.91-1.27)	82%	<0.01
		BR + BDQ VS BR + no other treatment	2	1.17(0.81-1.67)	81%	0.02	4	1.24(0.84-1.82)	89%	<0.01
	Subgroup analyses	BR + BDQ VS BR + placebo	2	1.07 (0.85-1.34)	0%	0.67	-	-	-	-
		BR + BDQ VS BR +SLID	-	-	-	-	1	**1.17(1.09-1.25)**	-	-
		BR + BDQ VS BR +SOC	1	0.82(0.70-0.94)	-	-	-	-	-	-
		BR + BDQ VS BR + DLM	-	-	-	-	4	1.08(0.91-1.27)	0%	0.40
The rate of cure at end of treatment	Main analyses		2	**1.60(1.13-2.26)**	0%	0.71	8	**1.86(1.23-2.83)**	93%	<0.01
		BR + BDQ VS BR + no other treatment	2	**1.60(1.13-2.26)**	0%	0.71	4	**2.19(1.47-3.28)**	90%	<0.01
	Subgroup analyses	BR + BDQ VS BR + placebo	-	-	-	-	-	-	-	-
		BR + BDQ VS BR +SLID	-	-	-	-	1	**1.17(1.09-1.25)**	-	-
		BR + BDQ VS BR +SOC	-	-	-	-	-	-	-	-
		BR + BDQ VS BR + DLM	-	-	-	-	3	**1.86(1.23-2.83)**	97%	<0.01
The rate of all-cause death at end of treatment	Main analyses		3	2.27(0.64-8.31)	27%	0.26	11	**0.68 (0.48-0.97)**	62%	<0.01
		BR + BDQ VS BR + no other treatment	-	-	-	-	7	**0.59(0.40-0.87)**	26%	0.23
	Subgroup analyses	BR + BDQ VS BR + placebo	2	**4.73(1.23-18.12)**	0	0.80	-	-	-	-
		BR + BDQ VS BR +SLID	-	-	-	-	-	-	-	-
		BR + BDQ VS BR +SOC	1	0.98(0.26-3.73)	-	-	-	-	-	-
		BR + BDQ VS BR + DLM	-	-	-	-	4	**0.85(0.48-0.97)**	75%	<0.01

Table S3. summary of results for the subgroup analyses, provided that excluded data for the imputed methods (continued).

**Outcomes**		**Type of intervention**	**RCT**				**NRS**			
			**No. of studies**	**Result (95%Cl)**	I^2	***P***	**No. of studies**	**Result (95%Cl)**	I^2	***P***
The rate of failure at end of treatment	Main analyses		4	0.58(0.35-0.88)	19%	0.30	10	**0.57(0.46-0.71)**	56%	0.02
		BR + BDQ VS BR + no other treatment	2	**0.35(0.16-0.77)**	0%	0.94	6	**0.53(0.39-0.71)**	51%	0.06
	Subgroup analyses	BR + BDQ VS BR + placebo	2	0.78(0.44-1.37)	0	0.44	-	-	-	-
		BR + BDQ VS BR +SLID	-	-	-	-	1	0.80(0.57-1.14)	-	-
		BR + BDQ VS BR +SOC	-	-	-	-	-	-	-	-
		BR + BDQ VS BR + DLM	-	-	-	-	3	**0.22(0.09-0.71)**	4%	0.02
The rate of lost to follow-up at end of treatment	Main analyses		3	1.99(0.61-6.51)	0%	0.69	11	0.84(0.58-1.21)		
		BR + BDQ VS BR + no other treatment	-	-	-	-	6	**0.65(0.43-0.97)**	25%	0.25
	Subgroup analyses	BR + BDQ VS BR + placebo	2	1.71(0.42-6.91)	0%	0.69	-	-	-	-
		BR + BDQ VS BR +SLID	-	-	-	-	1	**0.55(0.45-0.67)**	-	-
		BR + BDQ VS BR +SOC	1	2.02(0.62-6.57)	-	-	-	-	-	-
		BR + BDQ VS BR + DLM	-	-	-	-	4	1.56(0.95-2.57)	0%	0.45
Incidence of cardiotoxicity	Main analyses		8	**4.54(1.74-11.87)**	26%	0.24	6	**6.00(1.32-27.19)**	75%	<0.01
		BR + BDQ VS BR + no other treatment	3	**3.85(1.19-12.41)**	0%	0.53	4	**7.19(0.97-53.14)**	76%	<0.01
	Subgroup analyses	BR + BDQ VS BR + placebo	1	**34.05(2.13-543.62)**	-	-	-	-	-	-
		BR + BDQ VS BR +SOC	-	-	-	-	-	-	-	-
		BR + BDQ VS BR + DLM	-	-	-	-	2	5.67(0.18-177.61)	83%	0.02

Table S3. summary of results for the subgroup analyses, provided that excluded data for the imputed methods (continued).

**Outcomes**		**Type of intervention**	**RCT**				**NRS**			
			**No. of studies**	**Result (95%Cl)**	I^2	***P***	**No. of studies**	**Result (95%Cl)**	I^2	***P***
Incidence of hepatotoxicity	Main analyses		6	1.54(0.93-2.90)	0%	0.94	7	1.10(0.55-2.23)	66%	<0.78
	Subgroup analyses	BR + BDQ VS BR + no other treatment	3	1.42(0.67-3.01)	0%	0.92	5	1.32(0.52-3.32)	74%	<0.01
		BR + BDQ VS BR + placebo	1	5.16(0.26-103.26)	-	-	-	-	-	-
		BR + BDQ VS BR +SOC	1	2.50(0.92-2.85)	-	-	-	-	-	-
		BR + BDQ VS BR + DLM	-	-	-	-	2	1.10(0.55-2.23)	0%	0.71
Incidence of Grade 3-5 adverse events	Main analyses		6	**1.42(1.17-1.73)**	0%	0.86	3	1.56(0.28-8.63)	66%	0.05
	Subgroup analyses	BR + BDQ VS BR + no other treatment	-	-	-	-	3	1.56(0.28-8.63)	66%	0.05
		BR + BDQ VS BR + placebo	3	1.20(0.82-1.76)	0%	0.96	-	-	-	-
		BR + BDQ VS BR +SOC	1	**1.48(1.13-1.93)**	-	-	-	-	-	-
		BR + BDQ VS BR + DLM	-	-	-	-	-	-	-	-

BR: background regimen BDQ: bedaquiline; SOC: standard-of-care; SLID: second-line injectable drug; DLM: delamanid; RCT: randomized controlled trial; NRC: nonrandomized controlled trial; VS: versus

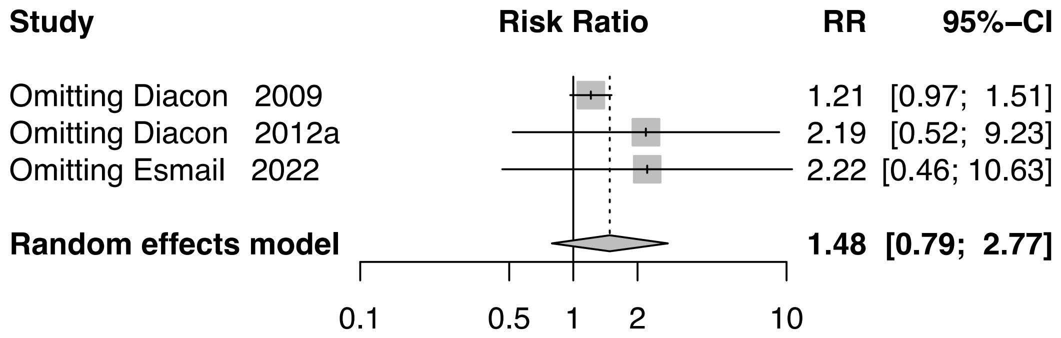

Figure S10 Sensitivity analysis of the rate of sputum culture conversion at 8 weeks in RCTs

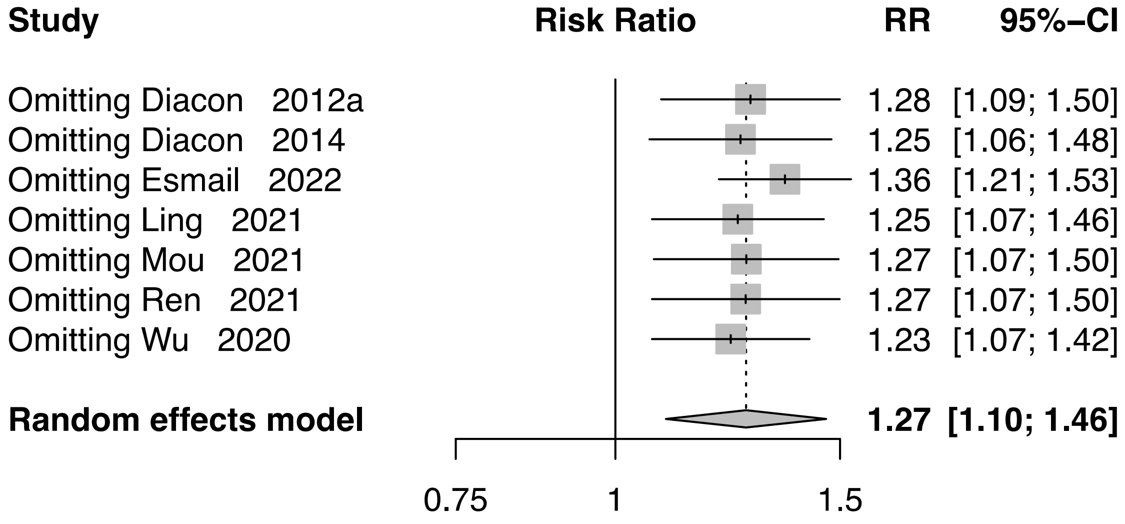

Figure S11 Sensitivity analysis of the rate of sputum culture conversion at 24 weeks in RCTs

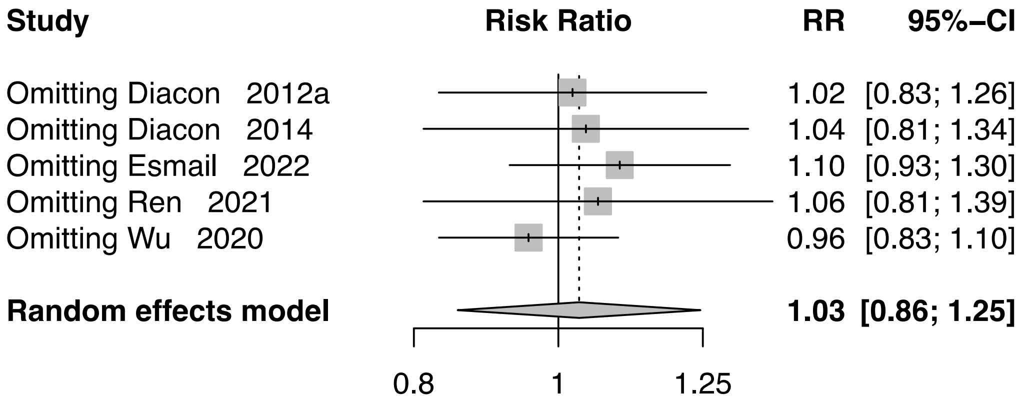

Figure S12 Sensitivity analysis of the rate of complete at end of the treatment in RCTs

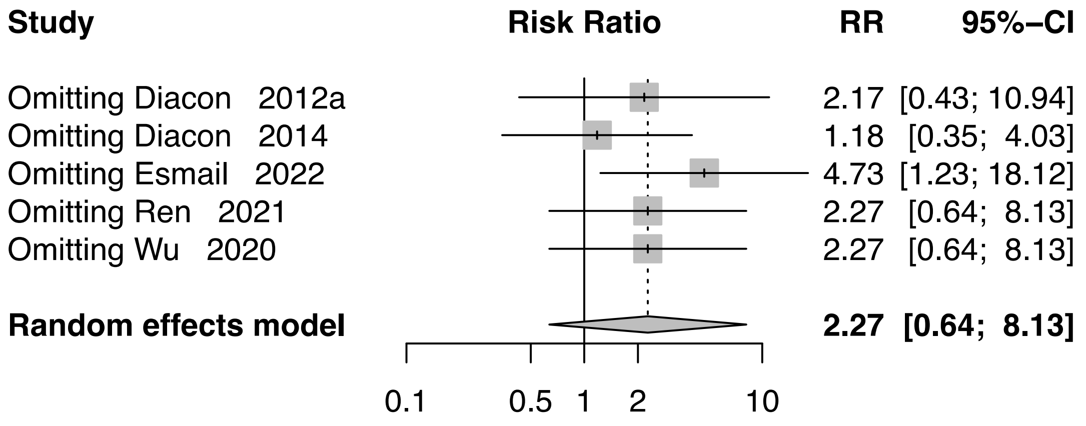

Figure S13 Sensitivity analysis of the rate of all-cause death at end of the treatment in RCTs

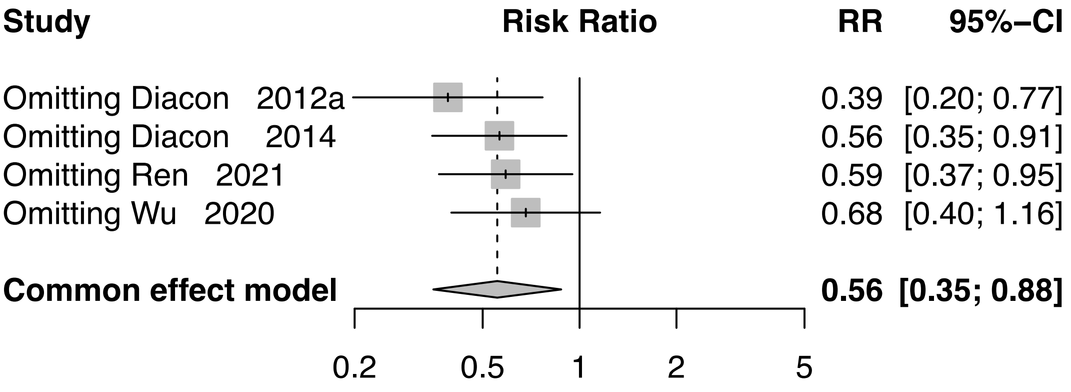

Figure S14 Sensitivity analysis of the failure rate at end of the treatment in RCTs

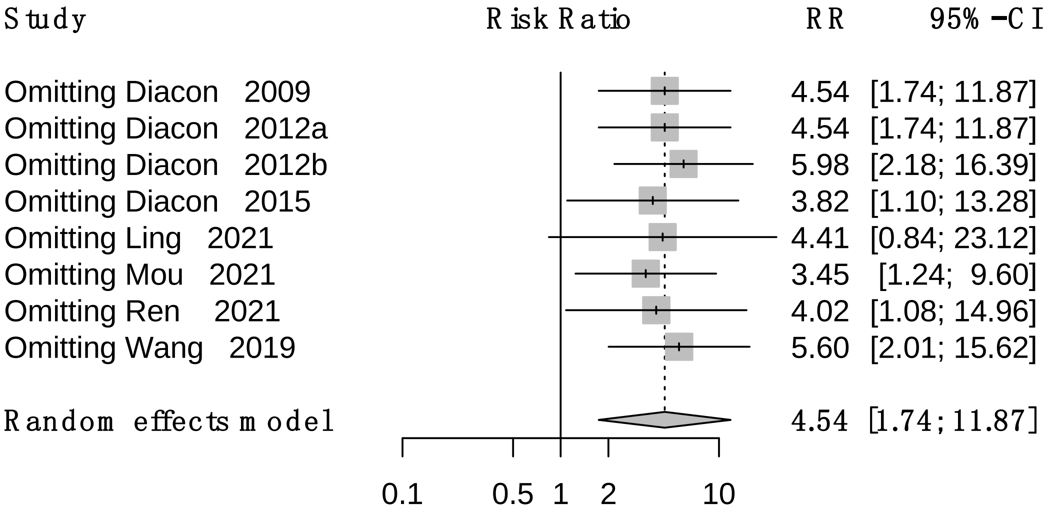

Figure S15 Sensitivity analysis of the incidence of cardiotoxicity in RCTs

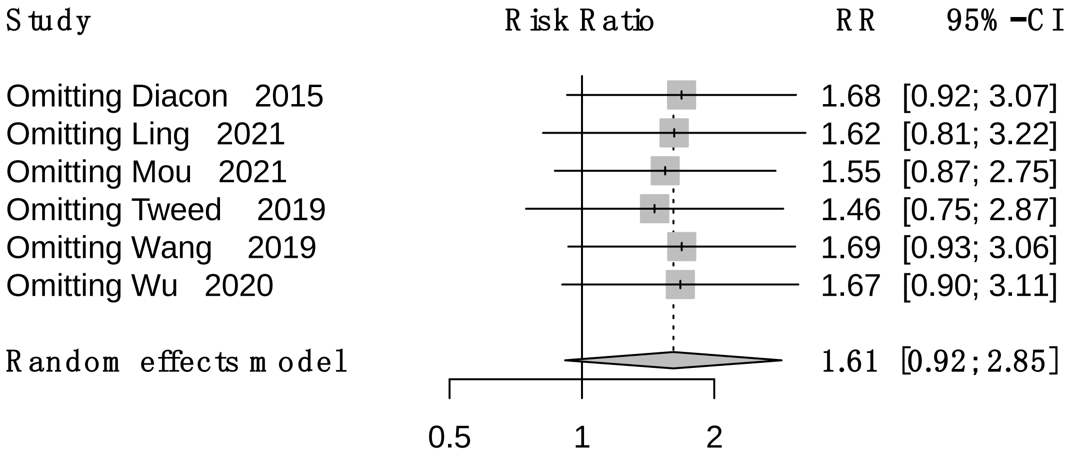

Figure S16 Sensitivity analysis of the incidence of hepatotoxicity in RCTs

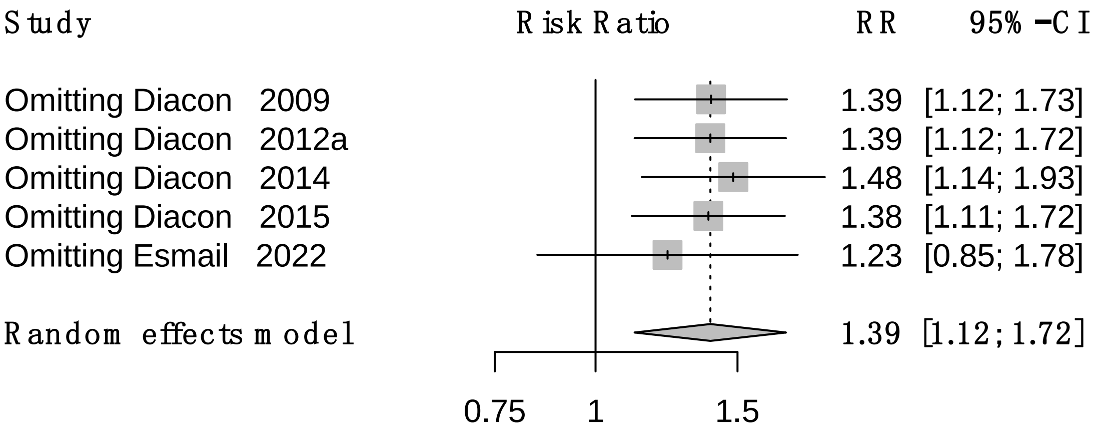

Figure S17 Sensitivity analysis of the incidence of grade 3-5 adverse events in RCTs
